# Synthesis of 3,4-Disubstituted Maleimide Derivatives via Phosphine-Catalyzed Isomerization of α-Succinimide-Substituted Allenoates Cascade γ′-Addition with Aryl Imines

**DOI:** 10.3390/ijms25136916

**Published:** 2024-06-24

**Authors:** Zhenzhen Gao, Xiaoming Zhou, Baoshen Nie, Hanchong Lu, Xiaotong Chen, Jiahui Wu, Xuekun Wang, Lei Li

**Affiliations:** School of Pharmaceutical Sciences, State Key Laboratory for Macromolecule Drugs and Large-Scale Manufacturing, Liaocheng University, Liaocheng 252059, China; 17861822319@163.com (X.Z.); 15806805721@163.com (B.N.); lhclhlhappyend@163.com (H.L.); 15621508513@163.com (X.C.); 18306595180@163.com (J.W.); wangxuekun@lcu.edu.cn (X.W.)

**Keywords:** phosphine-catalyzed, γ′-addition, isomerization, allenoates, 3,4-disubstituted maleimides

## Abstract

3,4-disubstituted maleimides find wide applications in various pharmacologically active compounds. This study presents a highly effective approach for synthesizing derivatives of 3,4-disubstituted maleimides through the direct isomerization of α-succinimide-substituted allenoates, followed by a cascade γ′-addition and aryl imines using PR_3_ as a catalyst. The resulting series of 3,4-disubstituted maleimides exhibited excellent stereoselectivities, achieving yields of up to 86%. To our knowledge, the phosphine-mediated γ′-addition reaction of allenoates is seldom reported.

## 1. Introduction

**Figure 1:**
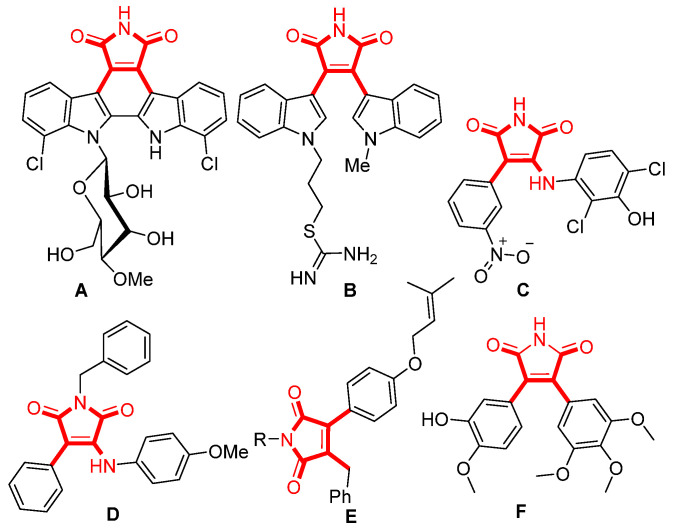
Some natural products and bioactive molecules. Compound **A**: rebeccamycin, an antibiotic; Compound **B**: indolylmaleimide derivative, inhibitor for cancer cells; Compound **C**: inhibitor of glycogen synthase kinase; Compound **D**: inhibitor for GSK-3; Compound **E**: an antifungal derived from the metabolite of the Serpula himantoides strain; Compound **F**: an antitumor agent.

Maleimide derivatives have been found to possess diverse biological activities. In fact, some 3,4-disubstituted maleimides are frequently found in natural products and bioactive molecules [1]. Figure 1 provides some examples of the different 3,4-disubstituted maleimide skeletons that occur. Rebeccamycin **A**, an antibiotic, has demonstrated noteworthy effectiveness against certain types of tumor cell lines [2]. Additionally, indolylmaleimide derivative **B** has the ability to inhibit the growth and movement of cancer cells, indicating its potential as an anticancer drug [3]. Compound **C**, a highly active inhibitor of glycogen synthase kinase, has been identified [4]. Recently, N-substituted maleimide **D** has been found to be an attractive inhibitor for GSK-3 and a potential treatment for Alzheimer’s disease [5]. **E** is an antifungal derived from the metabolite of the Serpula himantoides strain [6]. Additionally, 3,4-di(aryl)maleimide **F** is an antitumor agent that exhibits antitubulin activity [7]. As a result, the synthesis of these interesting molecular structures has presented a constant challenge to synthetic organic chemists.

**Scheme 1:**
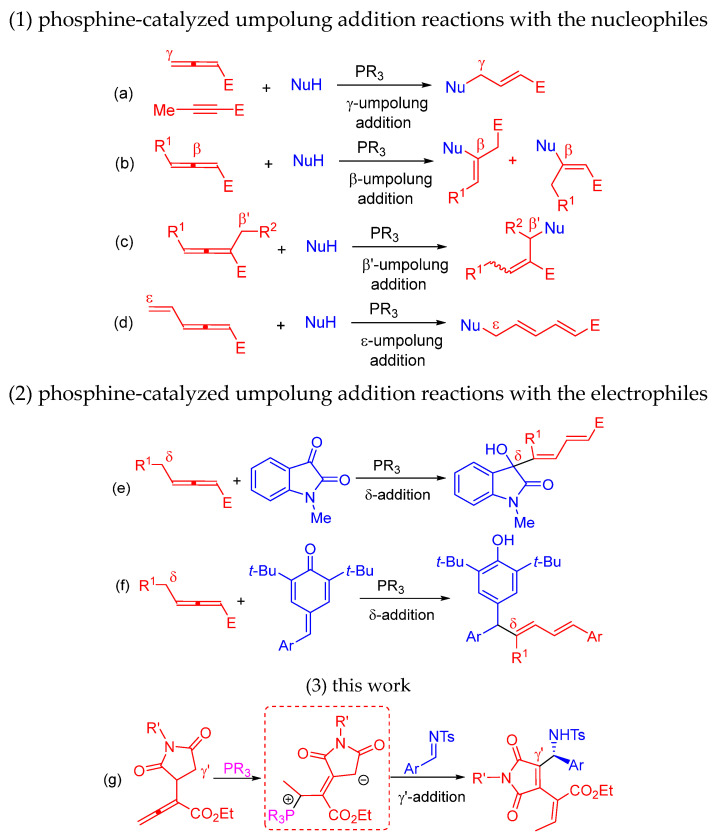
Various types of addition reactions to allenoates. (**a**): phosphine-catalyzed γ-umpolung addition of nucleophiles; (**b**): phosphine-catalyzed β-Michael additions to allenoates; (**c**): phosphine-catalyzed β′-umpolung addition; (**d**): phosphine-catalyzed ε-carbon 1,7-addition reactions; (**e**): phosphine-catalyzed δ-umpolung addition of isatin derivatives; (**f**): phosphine-catalyzed δ-umpolung addition of *para*-quinone methides; (**g**): phosphine-catalyzed isomerization cascade γ′-addition reaction.

Phosphine-catalyzed reactions have attracted significant interest over the past few decades [8,9,10,11,12,13,14,15,16,17] due to their ability to facilitate a variety of C-C and C-N bond formations in organic synthesis without introducing metal contamination into the organic products [18,19,20,21,22,23,24,25,26,27]. In 1994, Trost described the first example of a phosphine-catalyzed γ-umpolung addition of nucleophiles onto the γ-carbon of 2-butyrates (Scheme 1a) [28]. Subsequently, Lu utilized 2,3-allenoates for reacting with a nucleophile in the presence of PPh_3_, resulting in another γ-umpolung addition on the γ-carbon of the 2,3-allenoate (Scheme 1a) [29]. In the following years, Lu, Kwon, and Huang each demonstrated successful phosphine-catalyzed β-Michael additions to allenoates (Scheme 1b) [30,31,32,33,34,35,36]. In 2011, Kwon and Shi independently introduced α-alkyl allenoates and achieved a novel β′-umpolung addition for synthesizing functionalized acrylates (Scheme 1c) [37,38]. In 2020, Huang and colleagues employed vinyl allenoates to achieve the remote activation of the ε-carbon, leading to 1,7-addition reactions (Scheme 1d) [39]. Aside from nucleophile addition reactions, addition reactions of electrophiles with δ-substitute allenoates have also been reported. Prior studies have shown that most reactions between allenoates and electrophiles result in cyclizations. However, Xu and Li separately reported a notable phosphine-catalyzed δ-umpolung addition of isatin derivatives (Scheme 1e) or *para*-quinone methides (Scheme 1f) with δ-substitute allenoates [40,41]. Despite the recent advances, further diversification of allenoates is still necessary to foster the development of new addition reactions and versatile product motifs.

Our group has recently presented a novel set of α-succinimide-substituted allenoates, demonstrating the ability to undergo [4+2] annulation reactions with 1,1-dicyanoalkenes under phosphine catalysis, specifically involving the γ′-carbon of the allenoates [42]. However, as far as we know, the phosphine-mediated γ′-addition reaction of allenoates has been uncommon until now.

In this work, we introduce a new phosphine-catalyzed direct isomerization of α-succinimide-substituted allenoates, leading to cascade γ′-addition reactions with imine derivatives. It is conceivable that the attack of a phosphine on α-succinimide-substituted allenoates, along with subsequent proton transfers, could result in the formation of a putative intermediate where the remote γ′-carbon becomes nucleophilic, initiating fresh reactions. These reactions progress smoothly, yielding good to high yields of corresponding 3,4-disubstituted maleimide adducts under mild conditions (Scheme 1g).

## 2. Results and Discussion

The investigation began by combining N-tosyl (Ts) imine **1a** with α-succinimide-substituted allenoate **2a** in the presence of PBu_3_ (20 mol %) in DCM (2 mL) at room temperature, resulting in a 40% yield of **3a** (Table 1, entry 1). Further examination of the tertiary phosphine catalysts revealed that PMe_3_, MePPh_2_, EtPPh_2_, and PrPPh_2_ facilitated the addition reactions, yielding 37%, 43%, 55%, and 48%, respectively (Table 1, entries 2–5). Among these, EtPPh_2_ demonstrated the highest catalytic activity, producing the highest product yield (entry 4). The solvent exploration showed that DCE provided similar results to toluene (entries 6 and 8), while THF, CH_3_CN, and EtOAc yielded inferior results (entries 7, 9, and 10). In some phosphine-catalyzed reactions, protic reagents like phenol or benzoic acid were found to promote [1, 2], [1, 3], or [1, n] proton shifts and accelerate the reaction rates. Hence, various acidic protic additives were screened. The addition of the additives proved crucial to achieving higher yields (Table 1, entries 11–14). Using 30 mol % 3,5-(OH)_2_BzOH as an additive increased the yield to 73%. Adjusting the feeding ratio to **1a**:**2a** = 1:1.5 resulted in more efficient reaction progress and an 81% product yield (Table 1, entry 15). Consequently, the optimal reaction conditions were determined: DCM as the solvent, at room temperature, 20 mol % of the EtPPh_2_ as the catalyst, and 30 mol % 3,5-(OH)_2_BzOH as an additive.

Under the optimized conditions, we investigated the performance of various imines **1** and allenoates **2** in the γ′-addition reaction. The outcomes are outlined in Table 2. We observed that, when employing different substituted imines **1** (**1a**–**1n**) as substrates, the reaction proceeded effectively, yielding the desired product **3** in moderate to good yields (Table 2, entries 1–14). However, the presence of the electron-donating groups on the aromatic rings of the imines exhibited higher reactivity compared to the electron-withdrawing groups. Imine substrates **1b**–**1i** with electron-donating groups on the benzene ring yielded the desired products in moderate to good yields (72–86%) (Table 2, entries 2–9). Conversely, imines **1j**–**1n** with halogen substitutions on the benzene ring resulted in slightly lower yields (49–67%) (Table 2, entries 10–14). Unfortunately, when attempting to use alkyl imine (**1o**) as a reactant, no desired product was observed (Table 2, entry 15). Substituting the N-substituents in allenoates **2** with the 2-MeCH_2_C_6_H_4_ (**2b**), 4-MeCH_2_C_6_H_4_ (**2c**), and 4-OMeCH_2_C_6_H_4_ (**2d**) groups did not significantly alter the reactivity. Using these allenoates, the reaction proceeded smoothly to yield the desired products **3** in 59–64% yields (Table 2, entries 16–18).

**Figure 2:**
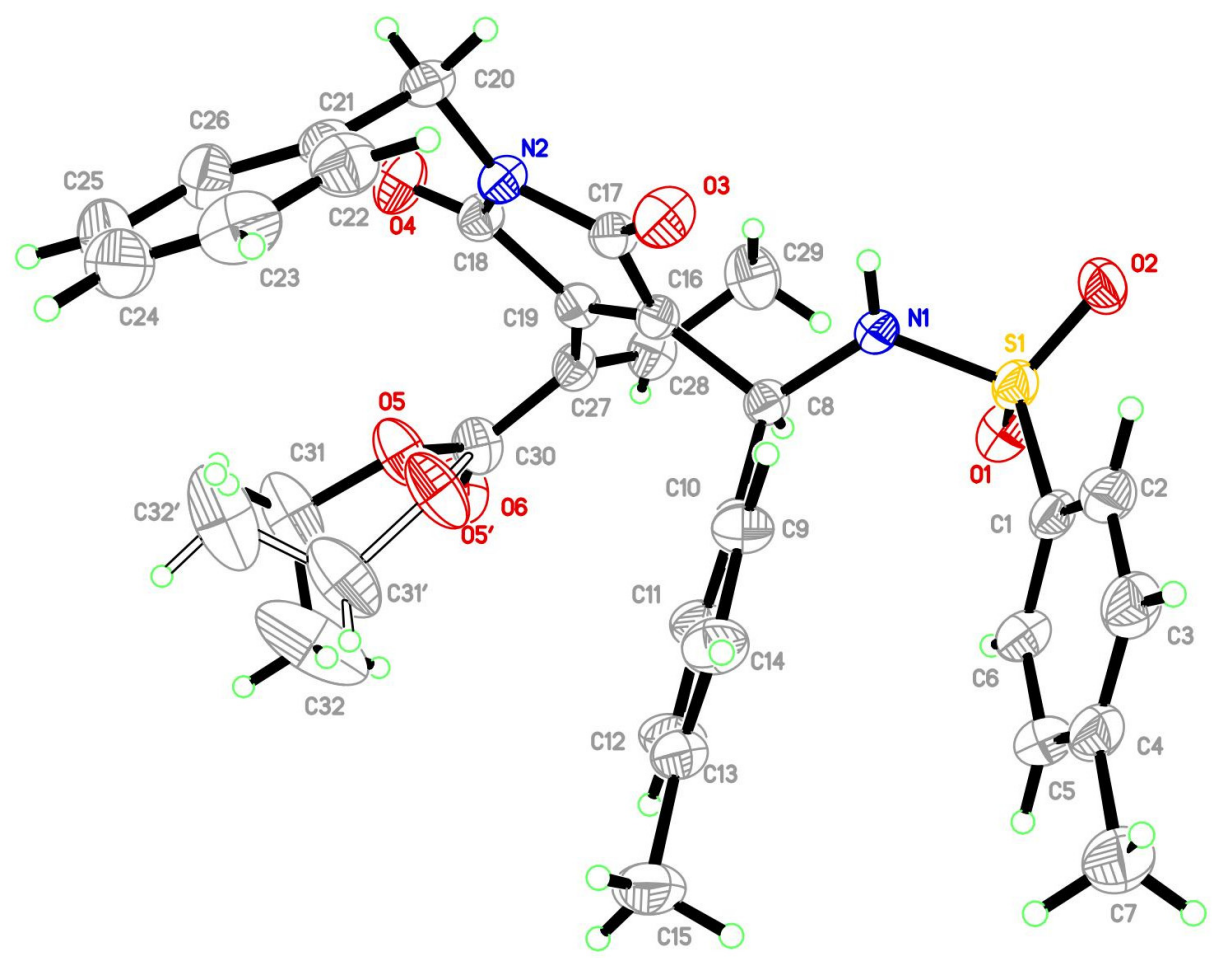
X-ray crystal structure of the **3da.** Grey balls: carbon atoms; red balls: oxygen atoms; blue balls: nitrogen atoms.

Moreover, the configurations and stereochemical properties of compound **3** were elucidated using NMR, high-resolution mass spectrometry (HRMS) data, and crystallographic analysis via X-ray diffraction (Figure 2). For detailed information on single crystal of **3da**, see Tables S1–S6 in the Supplementary Materials. The crystallographic data for **3da** have been submitted to the Cambridge Crystallographic Data Centre with deposition number CCDC 2258229 [43].

**Scheme 2:**
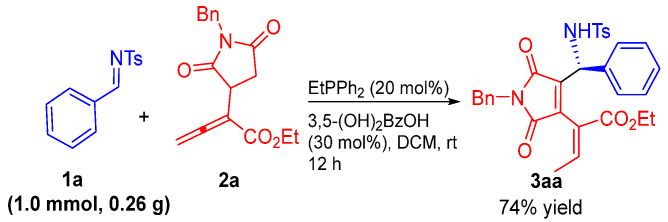
The scale-up reaction.

To demonstrate the applicability of our approach, we conducted a reaction at a larger scale. As shown in Scheme 2, under optimal conditions, the reaction between **1a** and α-succinimide-substituted allenoate **2a** proceeded smoothly, resulting in the production of the desired product **3aa** at the gram scale with no observable loss of yield.

**Scheme 3:**
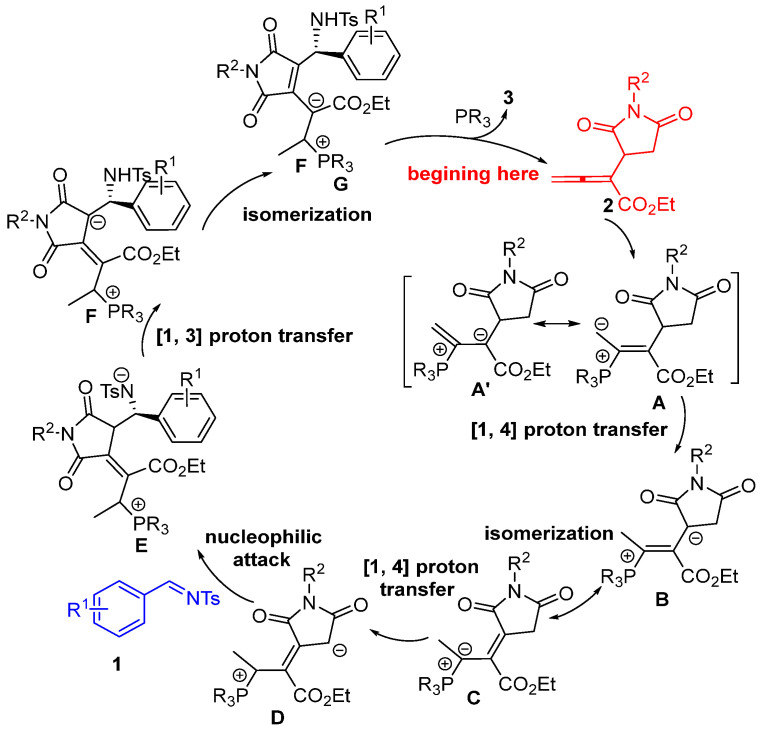
Proposed mechanism. The letter numbers **A**–**G** indicate possible intermediate structures in the plausible reaction mechanism.

A plausible reaction mechanism is presented in Scheme 3 [42]. This addition reaction begins with the nucleophilic addition of a phosphine to the allenoates **2**, forming zwitterionic intermediates (**A** ⟷ **A′**). Subsequently, proton transfer occurs, leading to the formation of intermediate **B**, followed by the generation of intermediate **D** through isomerization and proton transfer. Intermediate **D** then reacts with imines **1**, producing intermediate **E**. Subsequently, the intermediate **E** undergoes a series of H-transfers to form intermediate **G**. The elimination of PR_3_ from **G** yields product **3** and regenerates PR_3_ to complete the catalytic cycle.

## 3. Materials and Methods

Unless otherwise stated, all reagents were purchased from commercial suppliers and used without further purification. All solvents were filtered and dried according to standard procedures before use. All reactions were performed in dry glass vessels under nitrogen and magnetic stirring. The reaction was monitored by thin-layer chromatography (TLC) on silica precoated glass plates. The chromatogram was viewed under 254 nm UV light. Qingdao Marine flash silica gel (100–200 mesh) (Qingdao, China) was used for flash column chromatography. The ^1^H and ^13^C NMR spectra of CDCl_3_ were recorded using a 500 MHz NMR instrument. Melting point was measured using an X-4 digital micromelting point meter (Shanghai, China). Accurate mass measurements were conducted using Agilent instruments and ESI-MS technology (Santa Clara, CA, USA). X-ray crystallographic data were obtained using a Bruker D8 VENTURE instrument (Billerica, Germany).

### 3.1. General Procedure for the Synthesis of N-Tosyl Imines 1

Molecular Sieves (MS) 4Å was preactivated by microwave and dried under vacuum. A screw capped vial was charged with aldehyde (1.2 mmol), TsNH_2_ (1.0 mmol), and preactivated MS 4Å (1.0 g). Dried dichloromethane (3.0 mL) and pyrrolidine (6.22 μL, 0.10 mmol) were then added to the mixture. The resultant mixture was stirred at 60 °C for 24 h. The mixture was cooled to rt and filtered through a short pad of Celite^®^ from Tianjin Heowns (Tianjin, China). The organic phase was concentrated under reduced pressure, and the crude product was purified by crystallization (hexane/ethyl acetate system). The resulting solid was collected by filtration and then dried under vacuum.

### 3.2. General Procedure for the Synthesis of α-Succinimide-Substituted Allenoate 2

Maleimide (0.15 mmol), allene (0.30 mmol), DABCO (0.030 mmol), and 1,4-dioxane (1.0 mL) were added into a Schlenk tube. The reaction mixture was stirred at room temperature for 3 h or at room temperature for 3 h, the solvent was removed under reduced pressure, and the residue was purified by flash column chromatography (PE/EA = 4/1~2/1) to afford products **2a**–**2d**.

### 3.3. General Procedure for the Isomerization Cascade γ′-Addition Reaction

Under argon atmosphere, to a mixture of N-tosyl imines **1** (0.10 mmol), α-succinimide-substituted allenoate **2** (0.15 mmol), catalyst PR_3_ (20 mol %, 0.02 mmol), and the additive (30 mol %, 0.03 mmol) in a Schlenk tube, 2 mL of DCM was added at room temperature. The resulting mixture was stirred until the starting material was completely consumed (monitored by TLC) and then was concentrated to dryness. The residue was purified through flash column chromatography (EtOAc/PE) to afford the corresponding products **3**.

## 4. Conclusions

In conclusion, we have established a novel method for synthesizing functionalized 3,4-disubstituted maleimides in good yields. This reaction involves a phosphine-catalyzed isomerization cascade γ′-addition reaction between α-succinimide-substituted allenoates and aryl imine derivatives. Given the numerous biologically active natural products and industrially useful compounds containing 3,4-disubstituted maleimide components reported in the literature, our methodology offers a new protocol for efficiently synthesizing these compounds.

## Figures and Tables

**Table 1:** Reaction yields at different reaction conditions ^a^.

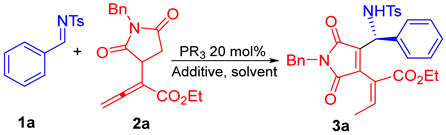					
Entry	PR_3_	Solvent	Additive	*t*/h	Yield (%) ^b^
1	PBu_3_	DCM	-	6	40
2	PMe_3_	DCM	-	6	37
3	MePPh_2_	DCM	-	6	43
4	EtPPh_2_	DCM	-	6	55
5	PrPPh_2_	DCM	-	6	48
6	EtPPh_2_	DCE	-	6	44
7	EtPPh_2_	THF	-	6	trace
8	EtPPh_2_	toluene	-	6	39
9	EtPPh_2_	CH_3_CN	-	6	trace
10	EtPPh_2_	EtOAc	-	6	23
11	EtPPh_2_	DCM	PhOH	6	60
12	EtPPh_2_	DCM	BzOH	6	68
13	EtPPh_2_	DCM	4-NO_2_BzOH	6	70
14	EtPPh_2_	DCM	3,5-(OH)_2_BzOH	6	73
15 ^c^	EtPPh_2_	DCM	3,5-(OH)_2_BzOH	8	81

^a^ Unless otherwise indicated, all reactions were carried out at room temperature using 0.10 mmol of **1a** and 0.12 mmol of **2a** in a solvent containing 20 mol % of the catalyst and 30 mol % of the additive. ^b^ Isolated yield. ^c^ **1a**:**2a** = 1:1.5.

**Table 2:** Substrate scope of phosphine-catalyzed γ′-addition reaction of α-succinimide-substituted allenoates ^a^.

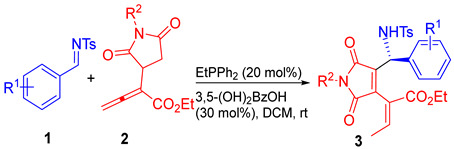				
Entry	R^1^ in 1	R^2^ in 2	3	Yield (%) ^b^
1	H (**1a**)	Bn (**2a**)	**3aa**	81
2	2-Me (**1b**)	Bn (**2a**)	**3ba**	83
3	3-Me (**1c**)	Bn (**2a**)	**3ca**	77
4	4-Me (**1d**)	Bn (**2a**)	**3da**	75
5	2-OMe (**1e**)	Bn (**2a**)	**3ea**	78
6	3-OMe (**1f**)	Bn (**2a**)	**3fa**	73
7	4-OMe (**1g**)	Bn (**2a**)	**3ga**	86
8	4-*i*Pr (**1h**)	Bn (**2a**)	**3ha**	72
9	2,5-2OMe (**1i**)	Bn (**2a**)	**3ia**	84
10 ^c^	2-F (**1j**)	Bn (**2a**)	**3ja**	51
11 ^c^	3-F (**1k**)	Bn (**2a**)	**3ka**	54
12	4-F (**1l**)	Bn (**2a**)	**3la**	67
13	4-Cl (**1m**)	Bn (**2a**)	**3ma**	65
14 ^c^	2-Br (**1n**)	Bn (**2a**)	**3na**	49
15 ^d^	Cyclohexyl (**1o**)	Bn (**2a**)	**3oa**	NR
16 ^c^	H (**1a**)	2-MeCH_2_C_6_H_4_ (**2b**)	**3ab**	59
17 ^c^	H (**1a**)	4-MeCH_2_C_6_H_4_ (**2c**)	**3ac**	65
18 ^c^	H (**1a**)	4-OMeCH_2_C_6_H_4_ (**2d**)	**3ad**	64

^a^ Unless otherwise indicated, all reactions were conducted at room temperature for 8 h using 0.10 mmol of compound **1** and 0.15 mmol of compound **2** in 2 mL DCM under the presence of 20 mol % of EtPPh_2_ and 30 mol % of 3,5-(OH)_2_C_6_H_3_CO_2_H. ^b^ Isolated yield. ^c^ 20 mol % of PBu_3_ was used instead of EtPPh_2_. ^d^ cyclohexyl instead of aryl.

## Data Availability

The data presented in this study are available on request from the authors.

## Supplementary Materials

The following supporting information can be downloaded at https://www.mdpi.com/article/10.3390/ijms25136916/s1. References [44,45] are mentioned in Supplementary Materials.
