# Supplementary material for: Synthesis of 3,4-Disubstituted Maleimide Derivatives via Phosphine-Catalyzed Isomerization of α-Succinimide-Substituted Allenoates Cascade γ′-Addition with Aryl Imines
Source: Int J Mol Sci. 2024 Jun 24;25(13):6916. doi: 10.3390/ijms25136916 (PMC11241244; doi:10.3390/ijms25136916)

Supporting Information

Synthesis of 3,4-Disubstituted Maleimide Derivatives via Phosphine-Catalyzed Isomerization of α-Succinimide Substituted Allenoates Cascade γ'-Addition with Aryl Imines

Zhenzhen Gao [1,*], Xiaoming Zhou [1], Baoshen Nie [1], Hanchong Lu [1], Xiaotong Chen [1], Jiahui Wu [1], Xuekun Wang [1], Lei Li [1,*]

[1] School of Pharmaceutical Sciences, State Key Laboratory for Macromolecule Drugs and Large-scale Manufacturing, Liaocheng University, Liaocheng, Shandong 252059, P. R. China. *Email:* gaozhenzhen@lcu.edu.cn; leili@lcu.edu.cn.

Table of Contents

General Information S2

General Procedure for Preparation of N-tosyl imines **1** S3

General Procedure for Preparation of α-Succinimide-Substituted Allenoate **2** S3

General Procedure for Preparation of Products **3** S4

^{1}H and ^{13}C NMR Spectra of New Compounds S14-S30

X-Ray Crystallographic Data of **3da** S31-S37

General Information

Unless otherwise stated, all reagents were purchased from commercial suppliers and used without further purification. All solvents were filtered and dried according to standard procedures ((1) Filter the solvents using vacuum filtration (2) Add anhydrous calcium chloride, stir and heat to reflux overnight (3) Collect the solvent by distillation (4) Store in a brown bottle and seal it) before use. All reactions were performed in dry glass vessels under nitrogen and magnetic stirring. The reaction was monitored by thin layer chromatography (TLC) on silica precoated glass plates. The chromatogram was viewed under 254 nm UV light. Qingdao Marine flash silica gel (100-200 mesh) (Qingdao, China) is used for flash column chromatography. The ^{1}H and ^{13}C NMR spectra of $CDCl_3$ were recorded using a 500 MHz NMR instrument. Melting point was measured using an X-4 digital micromelting point meter (Shanghai, China). Accurate mass measurements were made using Agilent instruments and ESI-MS technology (Santa Clara, CA, USA). X-ray crystallographic data were obtained using using a Bruker D8 VENTURE instrument (Billerica, Germany).

General Procedure for Synthesis of N-tosyl imines 1 [1]and α-Succinimide-Substituted Allenoate 2[2]

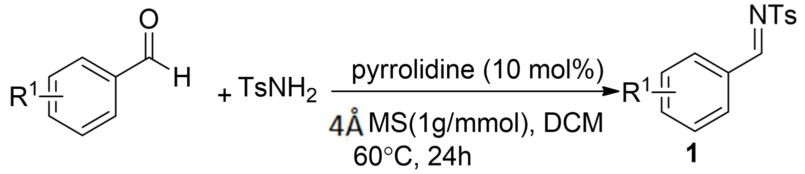

Molecular Sieves (MS) 4Å was preactivated by microwave and dried under vacuum. A screw capped vial was charged with aldehyde (1.2 mmol), $TsNH_2$ (1.0 mmol) and preactivated MS 4Å (1.0 g). Dried dichloromethane (3.0 mL) and pyrrolidine (6.22 μL, 0.10 mmol) were then added to the mixture. The resultant mixture was stirred at 60 °C for 24 h. The mixture was cooled to rt and filtered through a short pad of Celite®. The organic phase was concentrated under reduced pressure and the crude product was purified by crystallization (hexane/ethyl acetate system). The resulting solid was collected by filtration and then dried under vacuum.

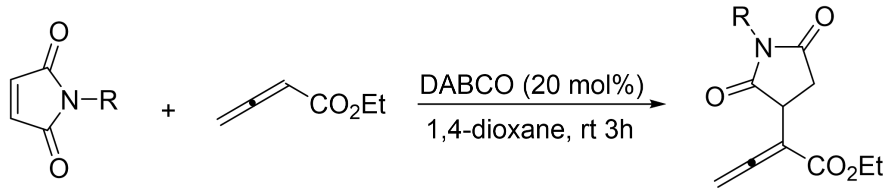

2a - 2d

Maleimide (0.15 mmol), allene (0.30 mmol), DABCO (0.030 mmol) , and 1,4-dioxane (1.0 mL) were added into a Schlenk tube. The reaction mixture was stirred at room temperature for 3 h or at room temperature for 3 h, the solvent was removed under reduced pressure, and the residue was purified by flash column chromatography (PE/EA= 4/1~2/1) to afford products **2a-2d** .

---

[1] S. Morales, F.G. Guijarro, J. G. Ruano, M. B. Cid, *J. Am. Chem. Soc.* **2014**, 136, 1082.

[2] Zhao Q Y, Pei C K, Guan X Y, et al. *Adv. Synth. Catal.* **2011**, 353, 1973 - 1979.

General Procedure for γ'-addition reaction of N-tosyl imines and α-Succinimide-Substituted Allenoate

Under argon atmosphere, to a mixture of N-tosyl imines **1** (0.10 mmol), α-succinimide substituted allenoate **2** (0.15 mmol), catalyst PR_3 (20 mol %, 0.02 mmol) and the additive (30 mol %, 0.03 mmol) in a Schlenk tube, 2 mL of DCM was added at room temperature. The resulting mixture was stirred until the starting material was completely consumed (monitored by TLC) and then was concentrated to dryness. The residue was purified through flash column chromatography (EtOAc / PE) to afford the corresponding products **3**.

Characterization Data of the Products 3

Ethyl (E)-2-(1-benzyl-4-(((4-methylphenyl)sulfonamido)(phenyl)methyl)-2,5-dioxo-2,5-dihydro-1H-pyrrol-3-yl)but-2-enoate (3aa)

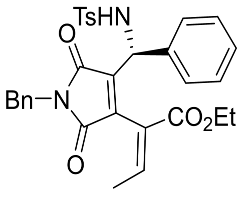

3aa

White solid, 81% yield. Purified by flash chromatography (12% EtOAc/PE). mp = 130.0- 131.0°C; ^{1}H NMR (500 MHz, $CDCl_3$) δ 7.48 (d, *J* = 7.9 Hz, 2H), 7.28 – 7.17 (m, 6H), 7.11 – 7.03 (m, 3H), 7.01 – 6.97 (m, 3H), 6.20 (s, 1H), 5.40 (d, *J* = 9.5 Hz, 1H), 4.55 (s, 2H), 4.12 – 3.84 (m, 2H), 2.25 (s, 3H), 1.54 (d, *J* = 5.6 Hz, 3H), 1.01 (t, *J* = 6.2 Hz, 3H). ^{13}C NMR (126 MHz, $CDCl_3$) δ 169.0, 167.7, 163.2, 146.2, 142.3, 139.5, 136.6, 135.8, 134.8, 134.2, 128.4, 127.9, 127.7, 127.7, 127.5, 127.1, 127.08, 126.9, 126.0, 125.95, 125.8, 125.7, 121.1, 60.5, 52.7, 40.9, 20.4, 15.4, 12.9. HRMS (ESI) calcd for $C_{31}H_{30}N_2O_6NaS$, $[M+Na]^+$ 581.1722, found 581.1727.

Ethyl (E)-2-(1-benzyl-4-(((4-methylphenyl)sulfonamido)(o-tolyl)methyl)-2,5-dioxo-2,5-dihydro-1H-pyrrol-3-yl)but-2-enoate (3ba)

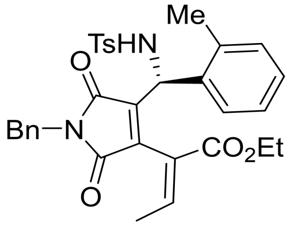

3ba

White solid, 83% yield. Purified by flash chromatography (12% EtOAc/PE). mp = 124- 125°C; ^{1}H NMR (500 MHz, $CDCl_3$) δ 7.51 (d, *J* = 8.0 Hz, 2H), 7.25 – 7.18 (m, 6H), 7.10 (dd, *J* = 18.3, 7.5 Hz, 2H), 7.00 (t, *J* = 8.1 Hz, 2H), 6.96 – 6.87 (m, 2H), 6.24 – 6.02 (m, 1H), 5.64 (d, *J* = 9.3 Hz, 1H), 4.60 – 4.45 (m, 2H), 4.12 – 3.87 (m, 2H), 2.26 (s, 3H), 2.06 (s, 3H), 1.41 (d, 3H), 1.03 (t, 3H). ^{13}C NMR (126 MHz, $CDCl_3$) δ 170.15, 168.8, 164.3, 147.2, 143.3, 140.6, 138.0, 137.7, 135.9, 135.0, 133.8, 129.4, 129.3, 128.8, 128.2, 127.9, 127.0, 126.8, 122.2, 61.5, 53.7, 41.9, 26.9, 21.5, 21.0, 16.4, 13.9. HRMS (ESI) calcd for $C_{32}H_{32}N_2O_6NaS$, $[M+Na]^+$ 595.1879, found 595.1883.

Ethyl (E)-2-(1-benzyl-4-(((4-methylphenyl)sulfonamido)(m-tolyl)methyl)-2,5-dioxo-2,5-dihydro-1H-pyrrol-3-yl)but-2-enoate (3ca)

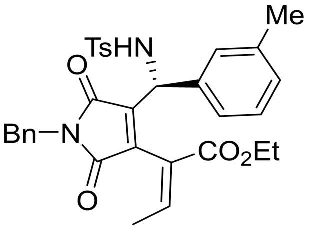

3ca

White solid, 77% yield. Purified by flash chromatography (12% EtOAc/PE). mp = 93.5- 94.5°C; ^{1}H NMR (500 MHz, $CDCl_3$) δ 7.48 (d, *J* = 8.2 Hz, 2H), 7.28 – 7.19 (m, 6H), 7.00 (d, *J* = 8.1 Hz, 2H), 6.96 (t, *J* = 7.6 Hz, 1H), 6.88 (d, *J* = 7.5 Hz, 1H), 6.79 (d, *J* = 7.7 Hz, 1H), 6.71 (s, 1H), 6.13 (d, *J* = 7.8 Hz, 1H), 5.35 (d, *J* = 9.6 Hz, 1H), 4.56 (s, 2H), 4.09 – 3.88 (m, 2H), 2.26 (s, 3H), 2.08 (s, 3H), 1.56 (d, *J* = 6.9 Hz, 3H), 1.03 (t, *J* = 7.0 Hz, 3H). ^{13}C NMR (126 MHz, $CDCl_3$) δ 169.1, 167.8, 163.3, 146.0, 144.3, 142.2, 139.5, 137.3, 136.7, 135.6, 134.9, 134.1, 128.3, 127.9, 127.8, 127.7, 127.7, 127.6, 127.2, 127.1, 126.9, 126.7, 126.5, 126.0, 122.9, 121.2, 60.8, 60.5, 52.8, 40.9, 28.7, 20.4, 20.2, 15.3, 13.0, 12.9. HRMS (ESI) calcd for $C_{32}H_{32}N_2O_6NaS$, $[M+Na]^+$ 595.1879, found 595.1881.

Ethyl (E)-2-(1-benzyl-4-(((4-methylphenyl)sulfonamido)(p-tolyl)methyl)-2,5-dioxo-2,5-dihydro-1H-pyrrol-3-yl)but-2-enoate (3da)

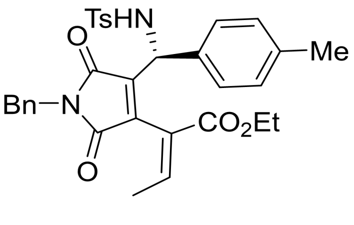

3da

White solid, 75% yield. Purified by flash chromatography (12% EtOAc/PE). mp = 96-97°C; ^{1}H NMR (500 MHz, $CDCl_3$) δ 7.55 (d, *J* = 8.3 Hz, 2H), 7.33 – 7.26 (m, 6H), 7.08 (d, *J* = 8.1 Hz, 2H), 6.94 (s, 4H), 6.22 – 6.05 (m, 1H), 5.41 (d, *J* = 9.6 Hz, 1H), 4.62 (s, 2H), 4.18 – 3.97 (m, 3H), 2.33 (s, 3H), 2.24 (s, 3H), 1.62 (d, *J* = 7.4 Hz, 3H), 1.09 (t, *J* = 11.8, 4.8 Hz, 3H). ^{13}C NMR (126 MHz, $CDCl_3$) δ 169.1, 167.6, 163.4, 145.9, 142.2, 140.0, 136.6, 134.9, 134.3, 129.7, 128.3, 127.7, 127.1, 126.9, 126.1,

126.0, 125.3, 121.3, 60.6, 40.9, 28.7, 20.4, 18.2, 15.2, 12.9. HRMS (ESI) calcd for $C_{32}H_{32}N_2O_6NaS$, $[M+Na]^+$ 595.1879, found 595.1873.

Ethyl (E)-2-(1-benzyl-4-((2-methoxyphenyl)((4-methylphenyl)sulfonamido)-methyl)-2,5-dioxo-2,5-dihydro-1H-pyrrol-3-yl)but-2-enoate (3ea)

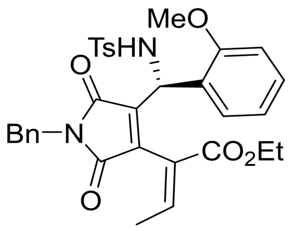

3ea

White solid, 78% yield. Purified by flash chromatography (12% EtOAc/PE). mp = 65-66°C; ^{1}H NMR (500 MHz, $CDCl_3$) δ 7.50 (d, J = 8.1 Hz, 2H), 7.25 – 7.17 (m, 6H), 7.05 (dd, J = 13.5, 7.7 Hz, 2H), 6.99 (d, J = 8.1 Hz, 2H), 6.64 (t, J = 7.5 Hz, 1H), 6.52 (d, J = 8.2 Hz, 1H), 5.69 (d, J = 9.9 Hz, 1H), 4.54 (s, 2H), 4.05 (dd, J = 14.3, 7.1 Hz, 2H), 3.43 (s, 3H), 2.24 (s, 3H), 1.53 (dd, J = 47.1 Hz, 3H), 1.46 – 1.38 (m, 1H), 1.04 (m, 3H). ^{13}C NMR (126 MHz, $CDCl_3$) δ 170.1, 168.6, 167.9, 154.8, 142.0, 139.9, 136.7, 135.1, 128.1, 127.9, 127.6, 127.1, 126.8, 126.0, 124.2, 121.5, 119.5, 109.0, 60.3, 59.4, 53.8, 40.7, 20.4, 15.0, 13.2, 13.0. HRMS (ESI) calcd for $C_{32}H_{32}N_2O_7NaS$, $[M+Na]^+$ 611.1828, found 611.1833.

Ethyl (E)-2-(1-benzyl-4-((3-methoxyphenyl)((4-methylphenyl)sulfonamido)-methyl)-2,5-dioxo-2,5-dihydro-1H-pyrrol-3-yl)but-2-enoate (3fa)

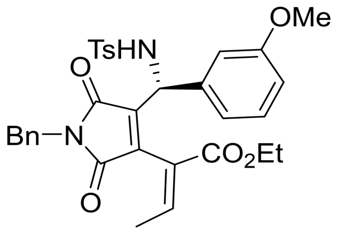

3fa

White solid, 73% yield. Purified by flash chromatography (12% EtOAc/PE). mp = 103.5- 104.5°C; ^{1}H NMR (500 MHz, $CDCl_3$) δ 7.49 (d, J = 8.3 Hz, 2H), 7.25 – 7.17 (m, 7H), 7.04 – 6.95 (m, 3H), 6.64 – 6.54 (m, 2H), 6.53 – 6.47 (m, 1H), 6.16 (s, 1H), 5.36 (d, J = 9.0 Hz, 1H), 4.56 (s, 2H), 4.04 (q, J = 8.6, 5.7 Hz, 2H), 3.56 (s, 3H), 2.26 (s, 3H), 1.57 (d, J = 7.0 Hz, 3H), 1.04 (t, 3H). ^{13}C NMR (126 MHz, $CDCl_3$) δ 170.1,

169.1, 167.7, 163.3, 158.8, 146.1, 142.3, 139.3, 137.2, 136.7, 134.8, 134.2, 128.7, 128.4, 127.9, 127.8, 127.7, 127.6, 127.5, 127.1, 126.9, 126.0, 121.1, 118.1, 112.9, 111.2, 60.5, 59.4, 54.1, 40.9, 20.4, 20.0, 15.4, 13.2, 12.9. HRMS (ESI) calcd for $C_{32}H_{32}N_2O_7NaS$, $[M+Na]^+$ 611.1828, found 611.1827.

Ethyl (E)-2-(1-benzyl-4-((4-methoxyphenyl)((4-methylphenyl)sulfonamido)-methyl)-2,5-dioxo-2,5-dihydro-1H-pyrrol-3-yl)but-2-enoate (3ga)

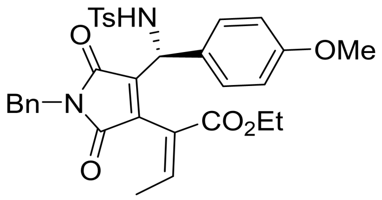

3ga

White solid, 86% yield. Purified by flash chromatography (12% EtOAc/PE). mp = 71.5- 72.5°C; ^{1}H NMR (500 MHz, $CDCl_3$) δ 7.47 (d, J = 8.3 Hz, 2H), 7.27 – 7.23 (m, 3H), 7.23 – 7.19 (m, 3H), 7.01 (d, J = 8.0 Hz, 2H), 6.94 – 6.88 (m, 2H), 6.63 – 6.53 (m, 2H), 6.08 (s, 1H), 5.32 (d, J = 9.5 Hz, 1H), 4.55 (s, 2H), 4.12 – 3.94 (m, 2H), 3.65 (s, 3H), 2.27 (s, 3H), 1.56 (d, J = 6.2 Hz, 3H), 1.10 – 0.99 (m, 3H). ^{13}C NMR (126 MHz, $CDCl_3$) δ 169.2, 167.8, 163.3, 158.4, 146.1, 142.2, 139.6, 136.7, 134.9, 133.8, 128.4, 127.9, 127.7, 127.2, 127.1, 126.9, 126.0, 121.2, 113.0, 60.5, 54.3, 52.4, 40.9, 20.4, 15.4, 12.9. HRMS (ESI) calcd for $C_{32}H_{32}N_2O_7NaS$, $[M+Na]^+$ 611.1828, found 611.1830.

Ethyl (E)-2-(1-benzyl-4-((4-isopropylphenyl)((4-methylphenyl)sulfonamido)-methyl)-2,5-dioxo-2,5-dihydro-1H-pyrrol-3-yl)but-2-enoate (3ha)

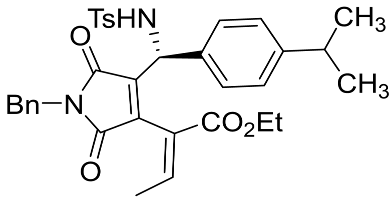

3ha

White solid, 72% yield. Purified by flash chromatography (12% EtOAc/PE). mp = 103- 104°C; ^{1}H NMR (500 MHz, $CDCl_3$) δ 7.44 (d, J = 8.3 Hz, 2H), 7.28 – 7.19 (m, 6H), 6.97 (d, J = 8.1 Hz, 2H), 6.89 – 6.88 (m, 3H), 6.17 (d, J = 7.4 Hz, 1H), 5.34 (d, J

= 9.6 Hz, 1H), 4.56 (s, 2H), 4.09 – 3.84 (m, 2H), 2.71 (dt, *J* = 13.8, 6.9 Hz, 1H), 2.24 (s, 3H), 1.57 (d, *J* = 6.3 Hz, 3H), 1.08 (s, 3H), 1.07 (s, 3H), 0.99 (t, *J* = 6.7 Hz, 3H). ^{13}C NMR (126 MHz, $CDCl_3$) δ 169.2, 167.8, 163.2, 148.0, 146.1, 142.1, 139.5, 136.6, 134.8, 133.9, 133.0, 128.3, 127.7, 127.1, 126.9, 126.0, 125.9, 125.7, 121.1, 60.4, 52.8, 40.9, 32.7, 22.9, 22.8, 20.4, 15.3, 12.9. HRMS (ESI) calcd for $C_{34}H_{36}N_2O_6NaS$, $[M+Na]^+$ 623.2186, found 623.2182.

Ethyl (E)-2-(1-benzyl-4-((2,5-dimethoxyphenyl)((4-methylphenyl)sulfonamido)-methyl)-2,5-dioxo-2,5-dihydro-1H-pyrrol-3-yl)but-2-enoate (3ia)

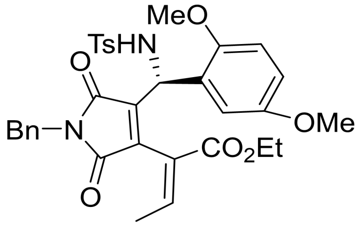

3ia

White solid, 84% yield. Purified by flash chromatography (12% EtOAc/PE). mp = 76-77°C; ^{1}H NMR (500 MHz, $CDCl_3$) δ 7.50 (d, *J* = 8.1 Hz, 2H), 7.24 – 7.21 (m, 2H), 7.21 – 7.16 (m, 3H), 7.13 – 7.02 (m, 1H), 6.99 (d, *J* = 8.1 Hz, 2H), 6.66 – 6.58 (m, 1H), 6.58 – 6.49 (m, 1H), 6.49 – 6.33 (m, 2H), 5.68 (d, *J* = 9.9 Hz, 1H), 4.54 (s, 2H), 4.12 – 3.89 (m, 2H), 3.54 (s, 3H), 3.40 (s, 3H), 2.24 (s, 3H), 1.64 – 1.48 (m, 3H), 1.11 – 0.97 (m, 3H). ^{13}C NMR (126 MHz, $CDCl_3$) δ 168.6, 167.9, 152.4, 149.0, 142.0, 139.7, 136.7, 135.1, 128.1, 127.8, 127.6, 127.1, 126.8, 126.0, 124.9, 121.5, 113.0, 110.0, 60.4, 54.6, 54.3, 40.8, 20.4, 15.1, 12.9. HRMS (ESI) calcd for $C_{33}H_{34}N_2O_8NaS$, $[M+Na]^+$ 641.1934, found 641.1934.

Ethyl (E)-2-(1-benzyl-4-((2-fluorophenyl)((4-methylphenyl)sulfonamido)methyl)-2,5-dioxo-2,5-dihydro-1H-pyrrol-3-yl)but-2-enoate (3ja)

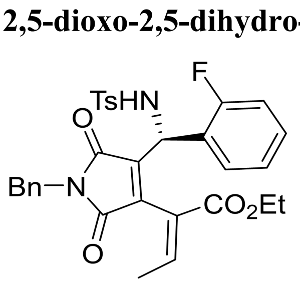

3ja

White solid, 51% yield. Purified by flash chromatography (12% EtOAc/PE). mp = 104.1- 105.1°C; ^{1}H NMR (500 MHz, $CDCl_3$) δ 7.60 (d, *J* = 7.8 Hz, 2H), 7.38 – 7.29 (m, 1H), 7.27 – 7.20 (m, 4H), 7.18 – 7.14 (m, 2H), 7.11 – 7.08 (m, 1H), 7.07 – 7.05 (m, 2H), 7.04 – 7.01 (m, 1H), 6.93 (s, 1H), 6.48 (s, 1H), 5.88 (d, *J* = 9.6 Hz, 1H), 4.55 (s, 2H), 4.15 – 3.98 (m, 2H), 2.28 (s, 3H), 1.45 (d, *J* = 5.6 Hz, 3H), 1.12 (t, *J* = 7.0 Hz, 3H). ^{13}C NMR (126 MHz, $CDCl_3$) δ 167.6, 145.7, 142.2, 139.4, 136.8, 134.9, 134.23, 128.5, 128.3, 128.2, 127.7, 127.0, 126.8, 126.1, 121.0, 60.7, 41.0, 28.7, 20.5, 15.3, 13.0. HRMS (ESI) calcd for $C_{31}H_{29}FN_2O_6NaS$, $[M+Na]^+$ 599.1628, found 599.1630.

Ethyl (E)-2-(1-benzyl-4-((3-fluorophenyl)((4-methylphenyl)sulfonamido)methyl)-2,5-dioxo-2,5-dihydro-1H-pyrrol-3-yl)but-2-enoate (3ka)

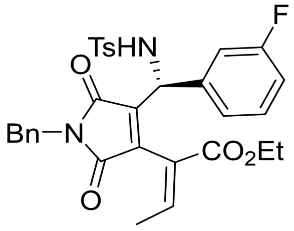

3ka

White solid, 54% yield. Purified by flash chromatography (12% EtOAc/PE). mp = 107.0- 108.0°C; ^{1}H NMR (500 MHz, $CDCl_3$) δ 7.50 (d, *J* = 8.2 Hz, 2H), 7.27 – 7.23 (m, 4H), 7.23 – 7.20 (m, 2H), 7.09 – 6.99 (m, 3H), 6.85 – 6.73 (m, 2H), 6.70 (d, *J* = 9.6 Hz, 1H), 6.19 (d, *J* = 7.6 Hz, 1H), 5.42 (d, *J* = 9.6 Hz, 1H), 4.56 (s, 2H), 4.12 – 3.96 (m, 2H), 2.27 (s, 3H), 1.59 (dd, *J* = 12.9, 7.3 Hz, 3H), 1.07 (t, *J* = 7.1 Hz, 3H). ^{13}C NMR (126 MHz, $CDCl_3$) δ 168.9, 167.5, 163.3, 162.7, 160.7, 146.3, 142.5, 138.9, 138.4, 138.4, 136.6, 134.7, 134.5, 129.3, 129.2, 128.4, 127.8, 127.2, 127.0, 125.9, 121.5, 121.0, 114.2, 114.0, 113.0, 112.9, 60.7, 52.0, 41.0, 28.7, 20.4, 15.4, 12.9. HRMS (ESI) calcd for $C_{31}H_{29}FN_2O_6NaS$, $[M+Na]^+$ 599.1628, found 599.1633.

Ethyl (E)-2-(1-benzyl-4-((4-fluorophenyl)((4-methylphenyl)sulfonamido)methyl)-2,5-dioxo-2,5-dihydro-1H-pyrrol-3-yl)but-2-enoate (3la)

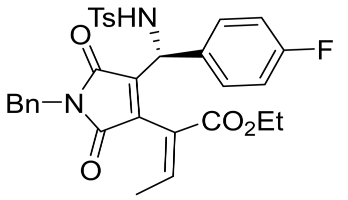

3la

White solid, 67% yield. Purified by flash chromatography (12% EtOAc/PE). mp = 100.5- 101.5°C; ^{1}H NMR (500 MHz, Chloroform-*d*) δ 7.47 (d, *J* = 8.2 Hz, 2H), 7.29 – 7.19 (m, 6H), 7.06 – 6.94 (m, 4H), 6.76 (t, *J* = 8.6 Hz, 2H), 6.15 (d, *J* = 9.6 Hz, 1H), 5.39 (d, *J* = 9.5 Hz, 1H), 4.56 (s, 2H), 4.12 – 3.92 (m, 2H), 2.27 (s, 3H), 1.57 (d, *J* = 7.3 Hz, 3H), 1.06 (t, *J* = 7.1 Hz, 3H). ^{13}C NMR (126 MHz, $CDCl_3$) δ 169.0, 167.6, 163.3, 162.3, 160.4, 146.3, 142.5, 139.2, 136.6, 134.7, 134.2, 131.7, 131.7, 128.4, 127.8, 127.72, 127.66, 127.4, 127.2, 127.0, 125.9, 125.6, 121.0, 114.7, 114.5, 60.6, 52.1, 41.0, 28.7, 20.4, 15.4, 12.9. HRMS (ESI) calcd for $C_{31}H_{29}FN_2O_6NaS$, $[M+Na]^+$ 599.1628, found 599.1631.

Ethyl (E)-2-(1-benzyl-4-((4-chlorophenyl)((4-methylphenyl)sulfonamido)methyl)-2,5-dioxo-2,5-dihydro-1H-pyrrol-3-yl)but-2-enoate (3ma)

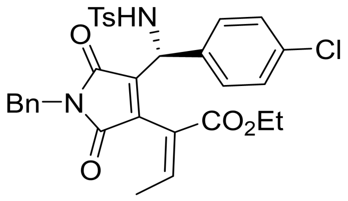

3ma

White solid, 65% yield. Purified by flash chromatography (12% EtOAc/PE). mp = 74.0- 75.0°C; ^{1}H NMR (500 MHz, $CDCl_3$) δ 7.46 (d, *J* = 8.3 Hz, 2H), 7.28 – 7.15 (m, 7H), 7.06 – 6.98 (m, 4H), 6.96 – 6.92 (m, 2H), 6.27 – 6.09 (m, 1H), 5.37 (d, *J* = 9.6 Hz, 1H), 4.55 (s, 2H), 4.13 – 3.93 (m, 2H), 2.28 (s, 3H), 1.58 (d, *J* = 6.5 Hz, 3H), 1.06 (t, *J* = 7.1 Hz, 3H). ^{13}C NMR (126 MHz, $CDCl_3$) δ 168.9, 167.5, 163.2, 146.4, 142.6, 138.9, 136.5, 134.7, 134.3, 133.1, 128.4, 127.8, 127.3, 127.2, 127.0, 125.9, 121.0, 60.6, 52.2, 41.0, 20.4, 15.5, 12.9. HRMS (ESI) calcd for $C_{31}H_{29}ClN_2O_6NaS$, $[M+Na]^+$ 615.1333, found 615.1340.

Ethyl (E)-2-(1-benzyl-4-((2-bromophenyl)((4-methylphenyl)sulfonamido)-

methyl)-2,5-dioxo-2,5-dihydro-1H-pyrrol-3-yl)but-2-enoate (3na)

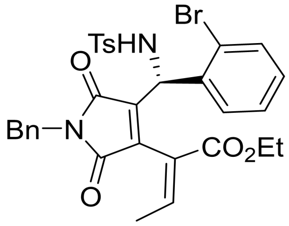

3na

White solid, 49% yield. Purified by flash chromatography (12% EtOAc/PE). mp = 134.5- 135.5°C; ^{1}H NMR (500 MHz, $CDCl_3$) δ 7.50 (d, *J* = 8.2 Hz, 2H), 7.27 – 7.23 (m, 4H), 7.23 – 7.20 (m, 2H), 7.09 – 6.99 (m, 3H), 6.85 – 6.73 (m, 2H), 6.70 (d, *J* = 9.6 Hz, 1H), 6.19 (d, *J* = 7.6 Hz, 1H), 5.42 (d, *J* = 9.6 Hz, 1H), 4.56 (s, 2H), 4.12 – 3.96 (m, 2H), 2.27 (s, 3H), 1.59 (dd, *J* = 12.9, 7.3 Hz, 3H), 1.07 (t, *J* = 7.1 Hz, 3H). ^{13}C NMR (126 MHz, $CDCl_3$) δ 167.6, 145.8, 142.2, 139.8, 136.9, 136.1, 134.9, 131.8, 128.4, 128.3, 127.7, 126.9, 126.8, 126.1, 121.0, 60.8, 41.0, 28.7, 20.5, 15.4, 13.1. HRMS (ESI) calcd for $C_{31}H_{29}BrN_2O_6NaS$, $[M+Na]^+$ 659.0827, found 659.0829.

Ethyl (E)-2-(1-(2-methylbenzyl)-4-(((4-methylphenyl)sulfonamido)(phenyl)-methyl)-2,5-dioxo-2,5-dihydro-1H-pyrrol-3-yl)but-2-enoate (3ab)

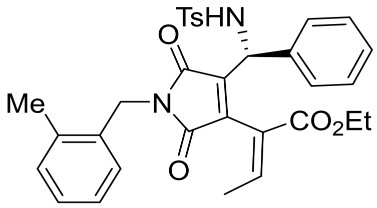

3ab

White solid, 59% yield. Purified by flash chromatography (12% EtOAc/PE). mp = 119.0- 120.0°C; ^{1}H NMR (500 MHz, $CDCl_3$) δ 7.49 (d, *J* = 8.2 Hz, 2H), 7.25 (q, *J* = 7.2 Hz, 1H), 7.13 – 7.04 (m, 6H), 7.04 – 6.98 (m, 5H), 6.16 (s, 1H), 5.41 (d, *J* = 9.5 Hz, 1H), 4.58 (s, 2H), 4.09 – 3.92 (m, 2H), 2.30 (s, 3H), 2.26 (s, 3H), 1.58 (d, *J* = 6.2 Hz, 3H), 1.03 (t, *J* = 6.9 Hz, 3H). ^{13}C NMR (126 MHz, $CDCl_3$) δ 169.2, 167.8, 163.2, 146.1, 142.3, 139.5, 136.6, 135.9, 134.9, 134.2, 132.6, 129.5, 128.4, 127.7, 127.1, 127.0, 126.8, 126.0, 125.8, 125.2, 121.1, 60.5, 52.7, 38.7, 28.7, 20.4, 18.3, 15.4, 12.9. HRMS (ESI) calcd for $C_{32}H_{32}N_2O_6NaS$, $[M+Na]^+$ 595.1879, found 595.1882.

Ethyl (E)-2-(1-(4-methylbenzyl)-4-(((4-methylphenyl)sulfonamido)(phenyl)-

methyl)-2,5-dioxo-2,5-dihydro-1H-pyrrol-3-yl)but-2-enoate

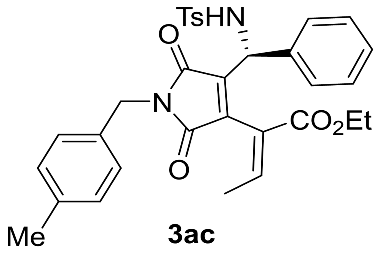

3ac

White solid, 65% yield. Purified by flash chromatography (12% EtOAc/PE). mp = 103.3- 104.3°C; ^{1}H NMR (500 MHz, $CDCl_3$) δ 7.48 (d, *J* = 8.2 Hz, 2H), 7.23 (q, *J* = 7.3 Hz, 1H), 7.12 – 7.07 (m, 4H), 7.07 – 7.03 (m, 3H), 7.00 (d, *J* = 8.1 Hz, 4H), 6.23 – 6.11 (m, 1H), 5.39 (d, *J* = 9.5 Hz, 1H), 4.51 (s, 2H), 4.09 – 3.93 (m, 2H), 2.26 (s, 6H), 1.55 – 1.51 (m, 3H), 1.03 (t, *J* = 7.0 Hz, 3H). ^{13}C NMR (126 MHz, $CDCl_3$) δ 169.1, 167.7, 163.3, 146.1, 142.3, 139.4, 136.7, 135.9, 134.2, 131.9, 128.4, 128.4, 127.7, 127.2, 127.1, 126.0, 125.8, 121.1, 60.5, 52.7, 40.7, 28.7, 20.4, 20.1, 15.4, 12.9. HRMS (ESI) calcd for $C_{32}H_{32}N_2O_6NaS$, $[M+Na]^+$ 595.1879, found 595.1885.

Ethyl (E)-2-(1-(4-methoxybenzyl)-4-(((4-methylphenyl)sulfonamido)(phenyl)-methyl)-2,5-dioxo-2,5-dihydro-1H-pyrrol-3-yl)but-2-enoate (3ad)

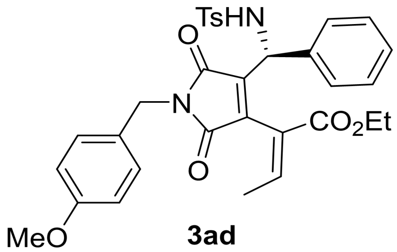

3ad

White solid, 64% yield. Purified by flash chromatography (12% EtOAc/PE). mp = 126.5- 127.5°C; ^{1}H NMR (500 MHz, $CDCl_3$) δ 7.48 (d, *J* = 8.3 Hz, 2H), 7.26 – 7.20 (m, 1H), 7.18 – 7.13 (m, 2H), 7.10 – 7.03 (m, 3H), 7.02 – 6.95 (m, 4H), 6.77 (dd, *J* = 6.7, 4.8 Hz, 2H), 6.19 (d, *J* = 7.8 Hz, 1H), 5.39 (d, *J* = 9.6 Hz, 1H), 4.49 (s, 2H), 4.10 – 3.92 (m, 2H), 3.72 (s, 3H), 2.25 (s, 3H), 1.53 (d, *J* = 6.8 Hz, 3H), 1.02 (t, *J* = 7.0 Hz, 3H). ^{13}C NMR (126 MHz, $CDCl_3$) δ 169.1, 167.8, 163.3, 158.3, 146.1, 142.3, 139.4, 136.6, 135.8, 134.1, 128.7, 128.4, 127.7, 127.1, 126.0, 125.8, 121.1, 113.1, 60.5, 54.3, 52.7, 40.4, 20.4, 15.4, 12.9. HRMS (ESI) calcd for $C_{32}H_{32}N_2O_7NaS$, $[M+Na]^+$ 611.1828, found 611.1832.

^{1}H NMR and ^{13}C NMR Spectra

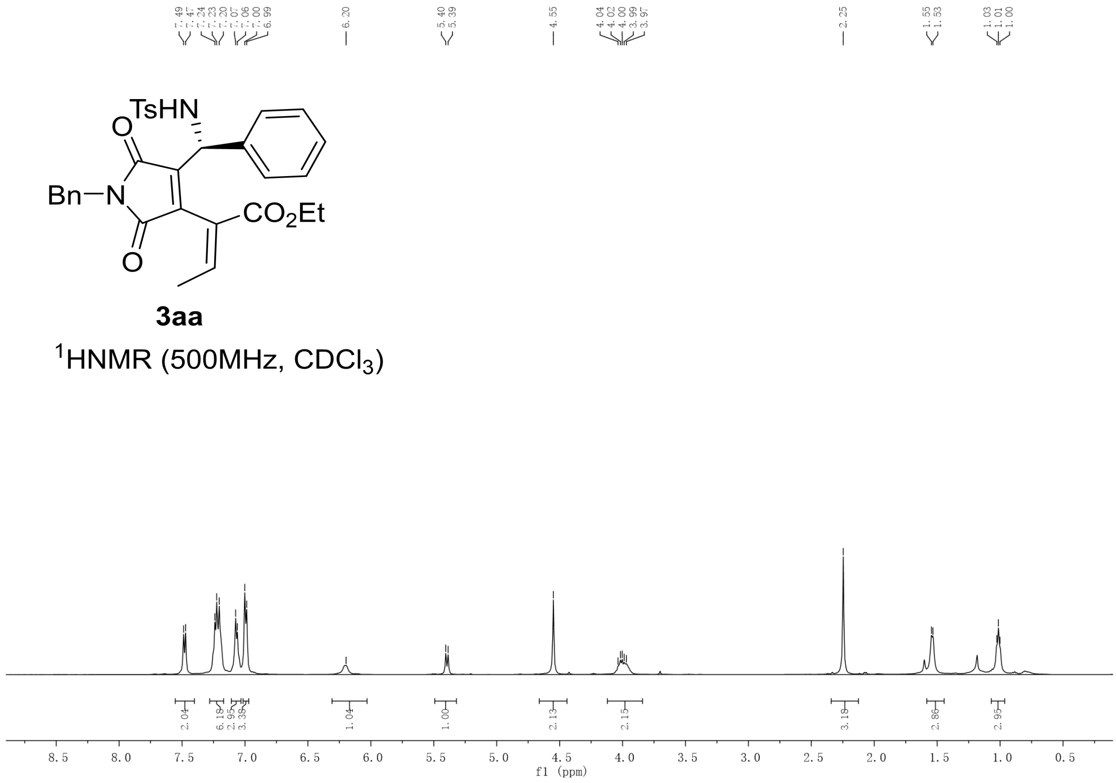

3aa

^{1}HNMR (500MHz, $CDCl_3$)

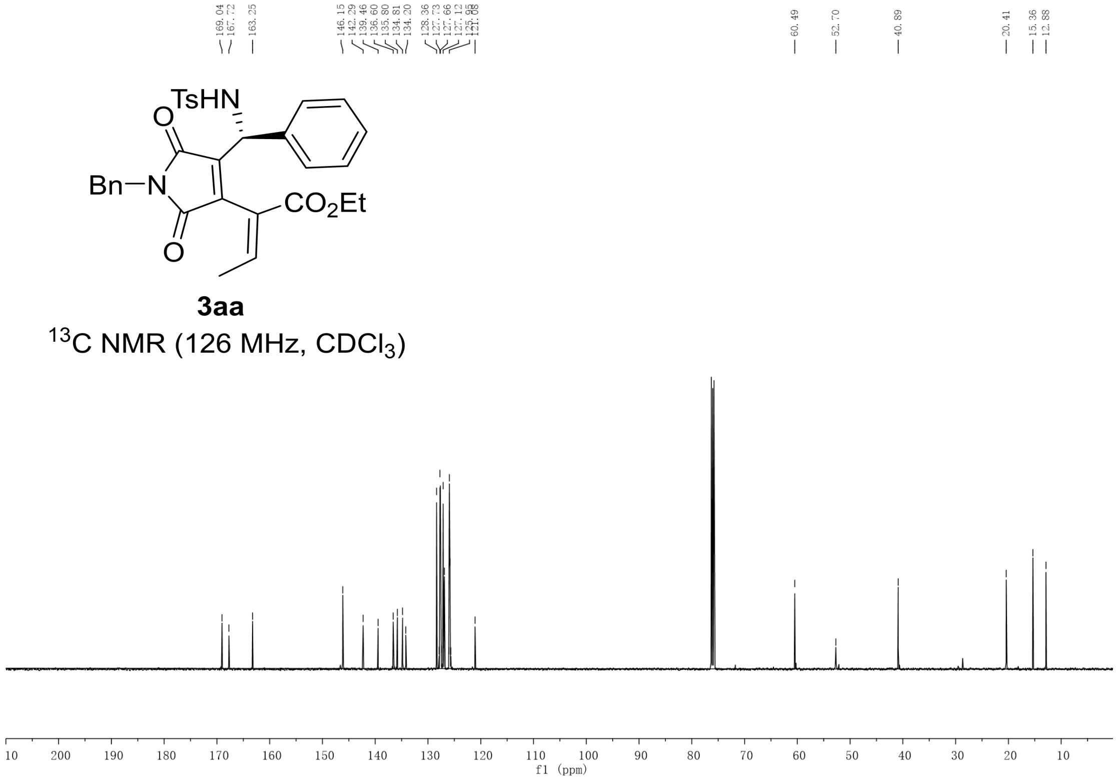

3aa

^{13}C NMR (126 MHz, $CDCl_3$)

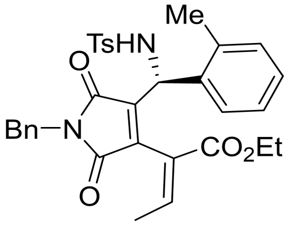

3ba

^{1}HNMR (500MHz, $CDCl_3$)

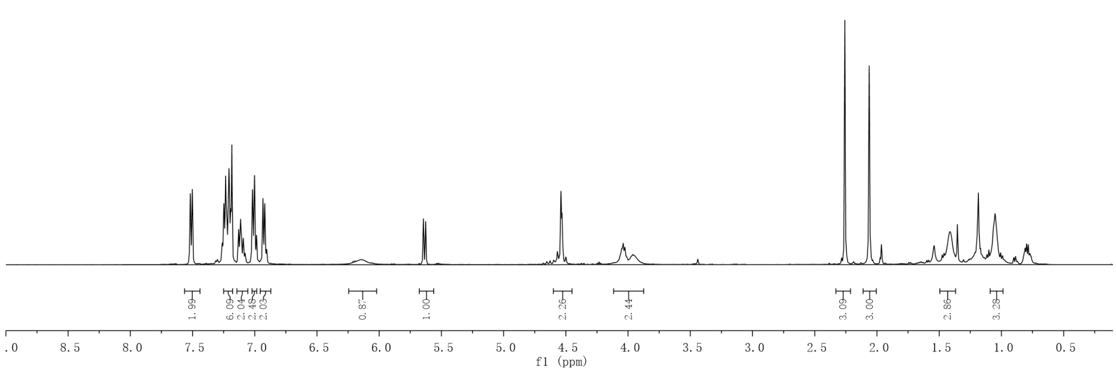

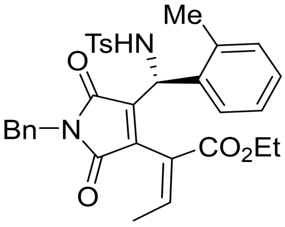

3ba

^{13}C NMR (126 MHz, $CDCl_3$)

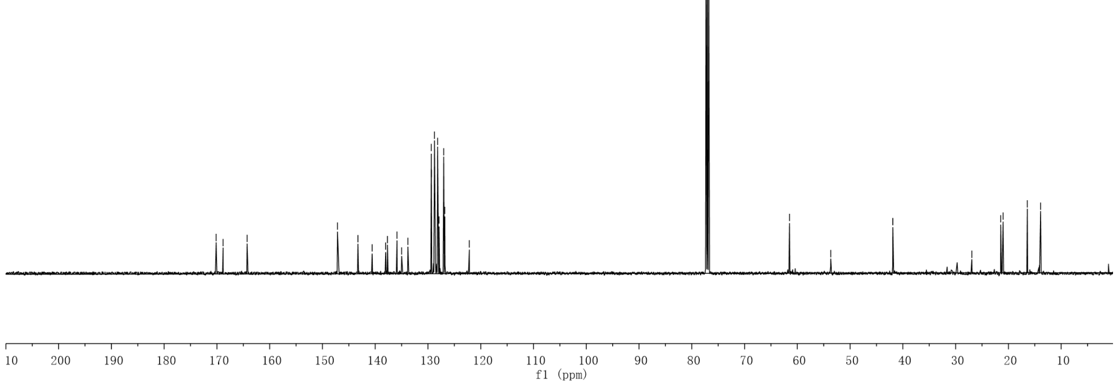

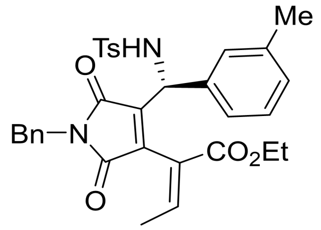

3ca

^{1}HNMR (500MHz, $CDCl_3$)

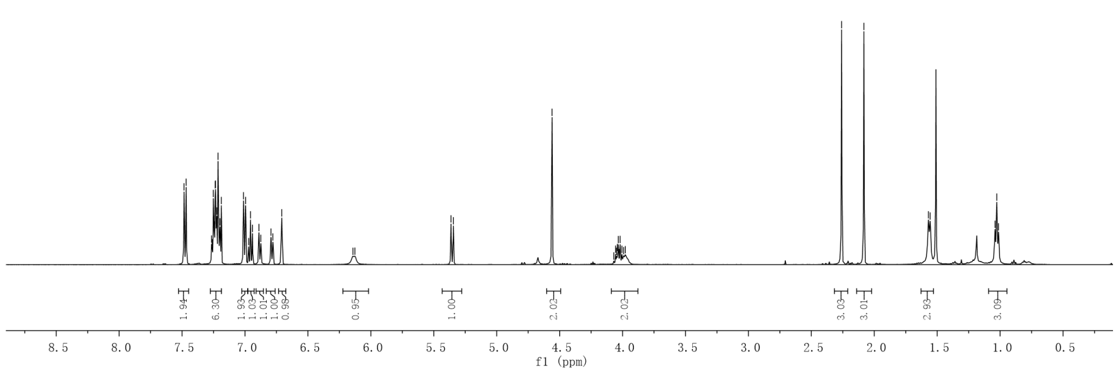

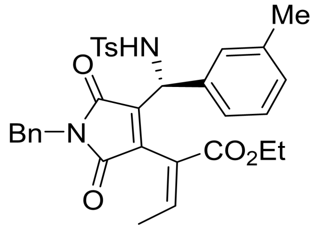

3ca

^{13}C NMR (126 MHz, $CDCl_3$)

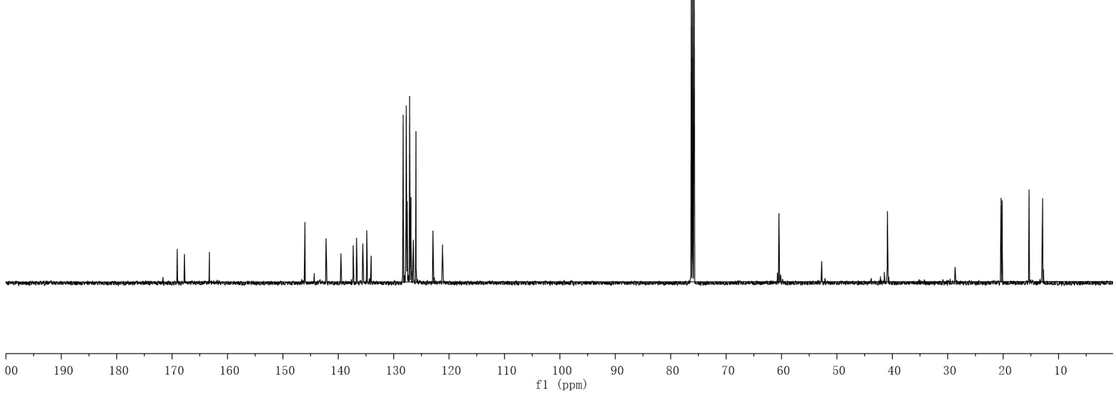

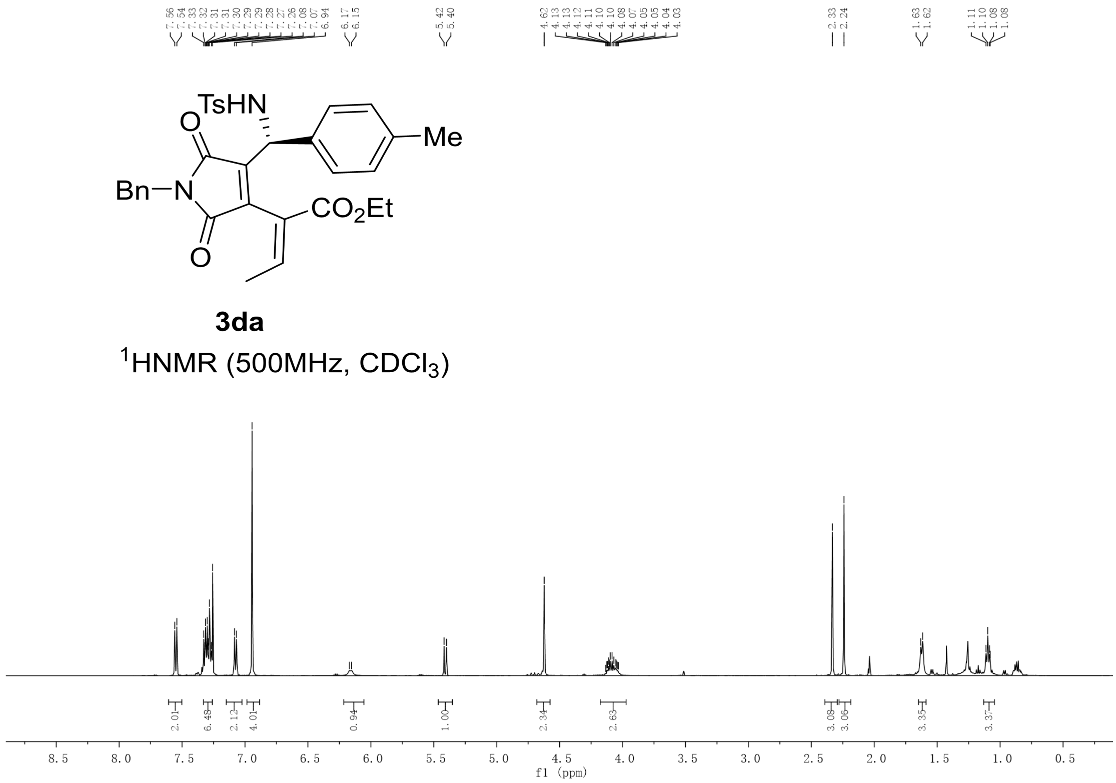
TsHN
Me
Bn
N
O
CO2Et
3da
1HNMR (500MHz, CDCl3)
f1 (ppm)

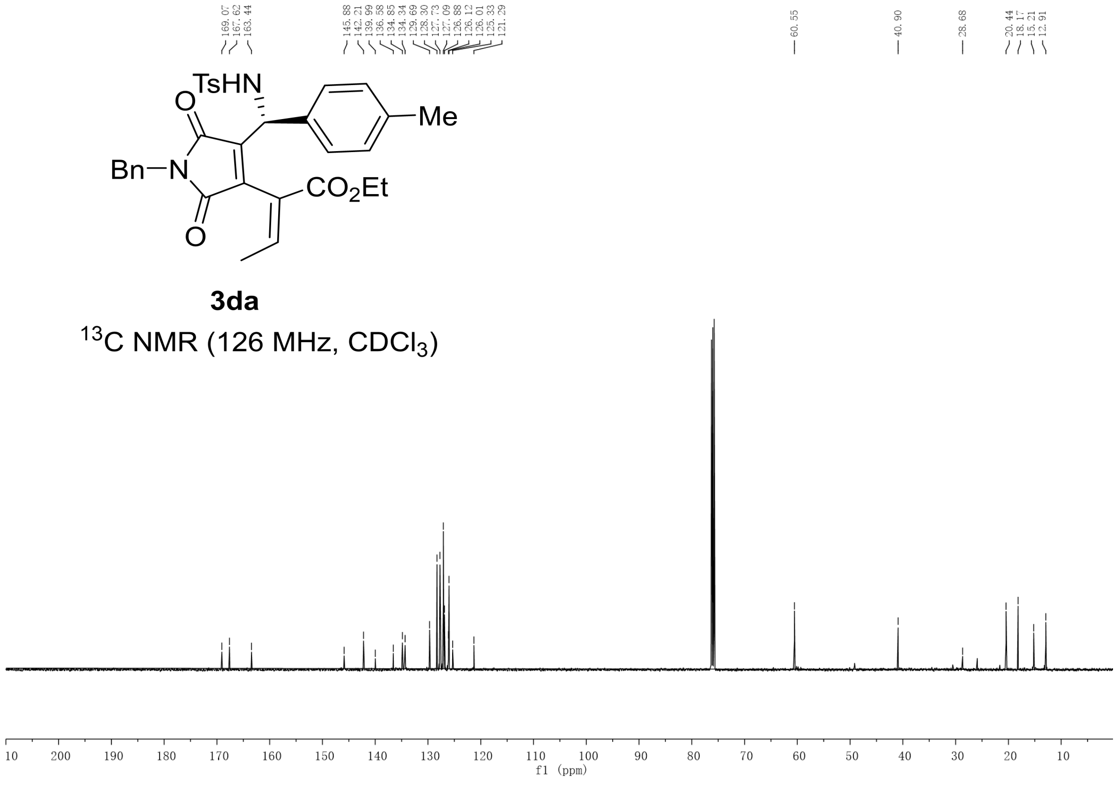
TsHN
Me
Bn
N
O
CO2Et
3da
13C NMR (126 MHz, CDCl3)
f1 (ppm)

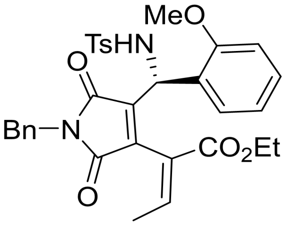

3ea

^{1}HNMR (500MHz, $CDCl_3$)

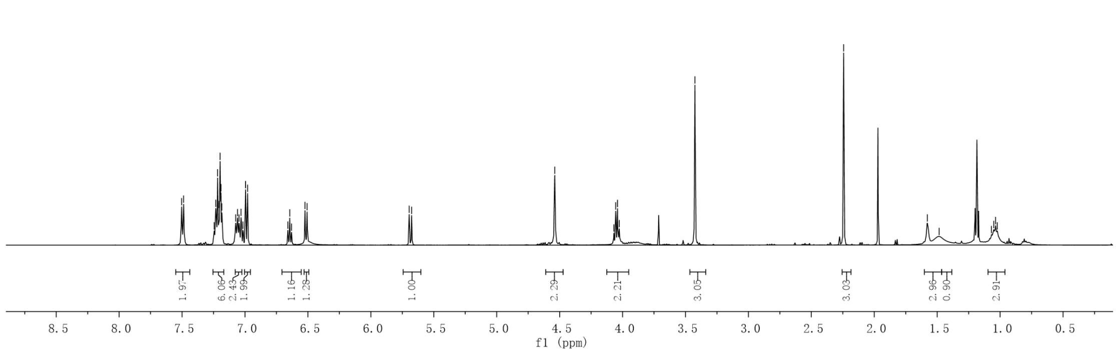

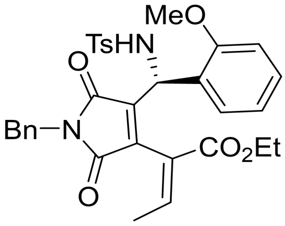

3ea

^{13}C NMR (126 MHz, $CDCl_3$)

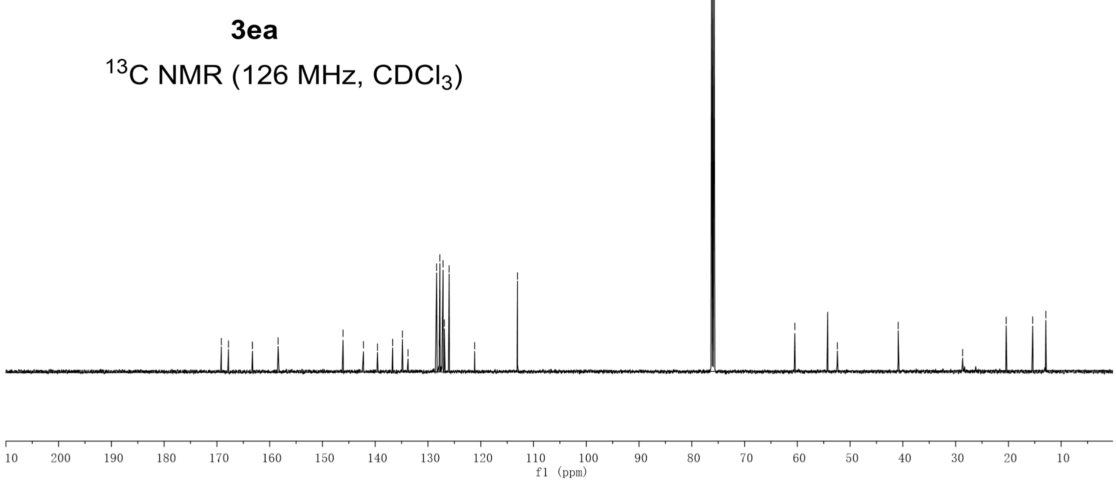

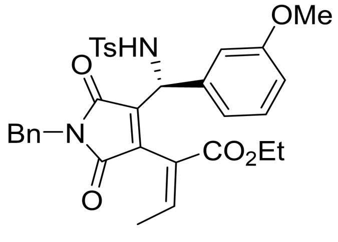

3fa

^{1}HNMR (500MHz, $CDCl_3$)

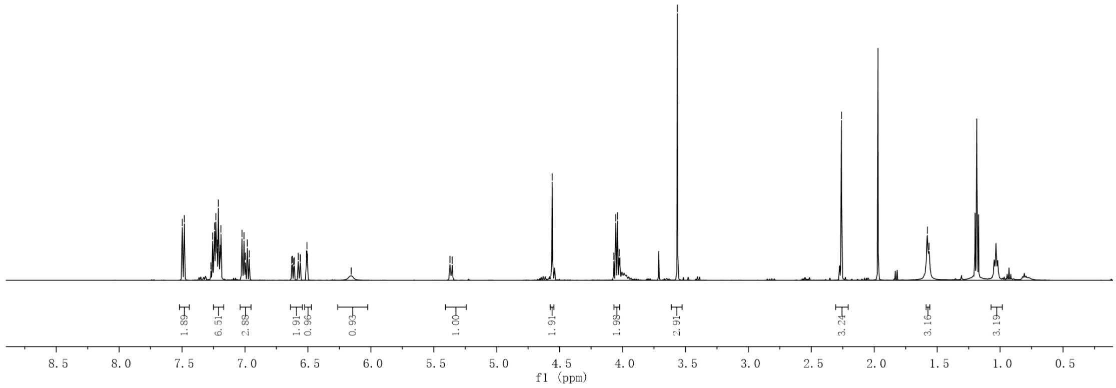

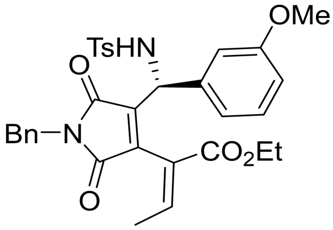

3fa

^{13}C NMR (126 MHz, $CDCl_3$)

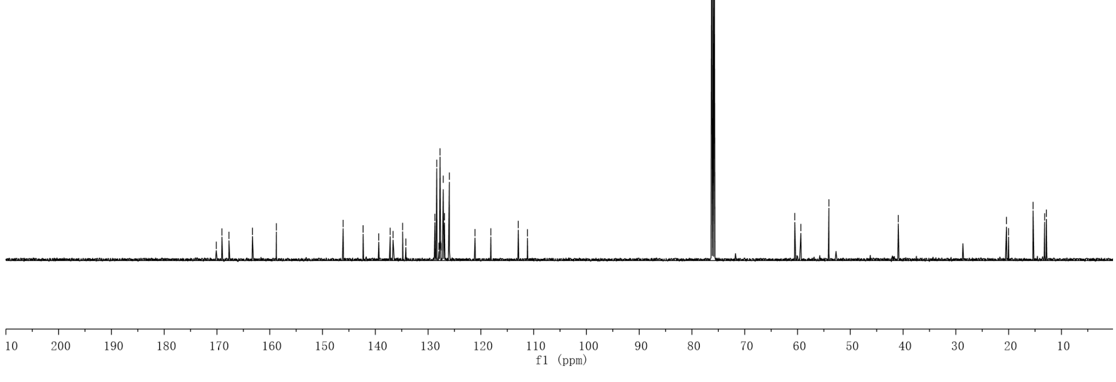

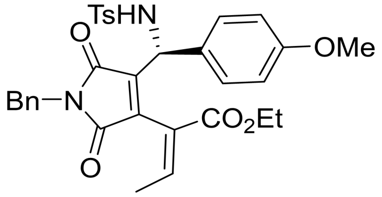

3ga

^{1}HNMR (500MHz, $CDCl_3$)

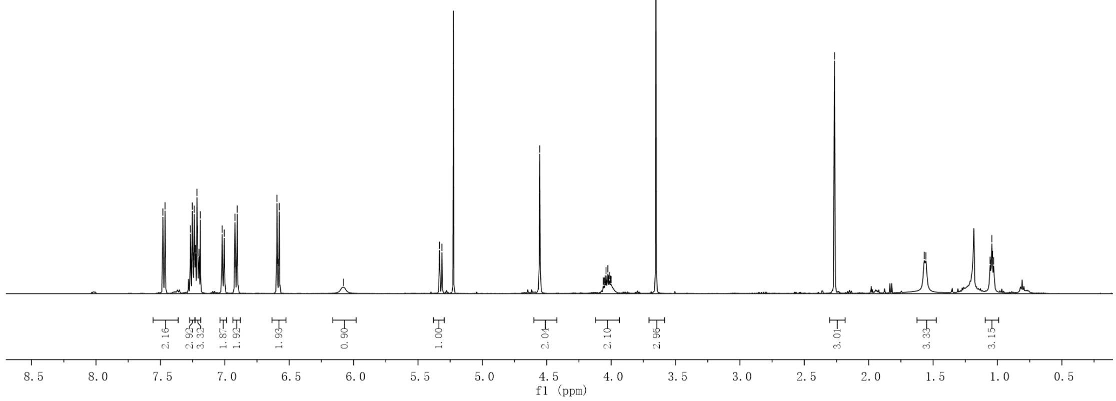

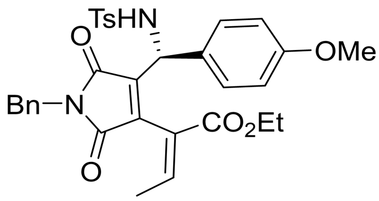

3ga

^{13}C NMR (126 MHz, $CDCl_3$)

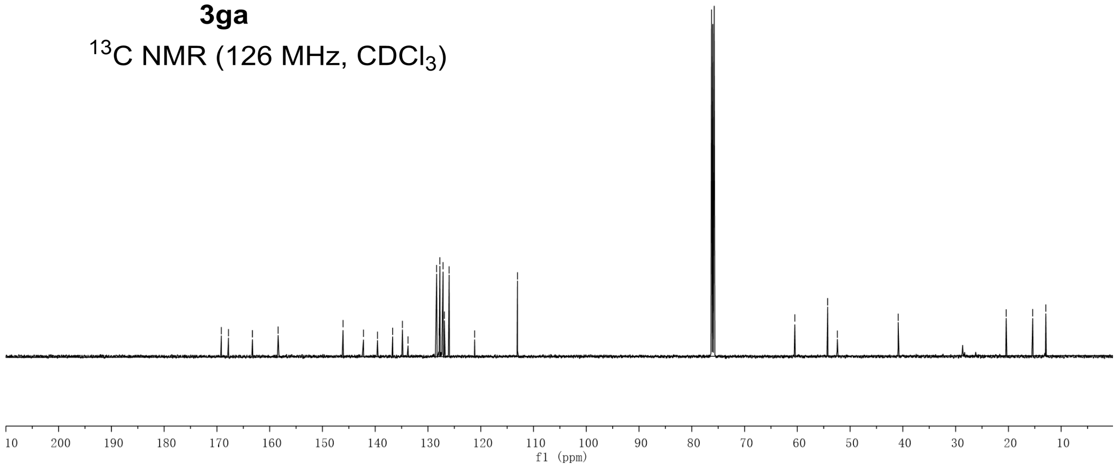

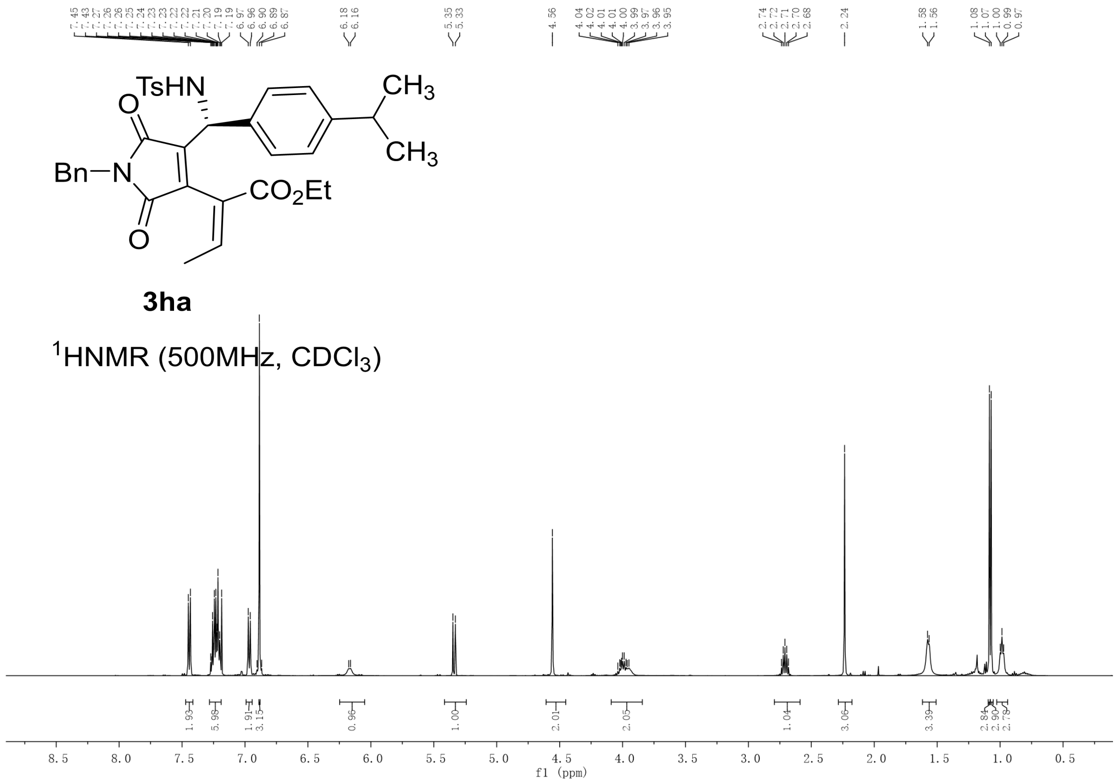
TsHN
O
Bn—N
CO2Et
O
CH3
CH3
3ha
1HNMR (500MHz, CDCl3)
f1 (ppm)

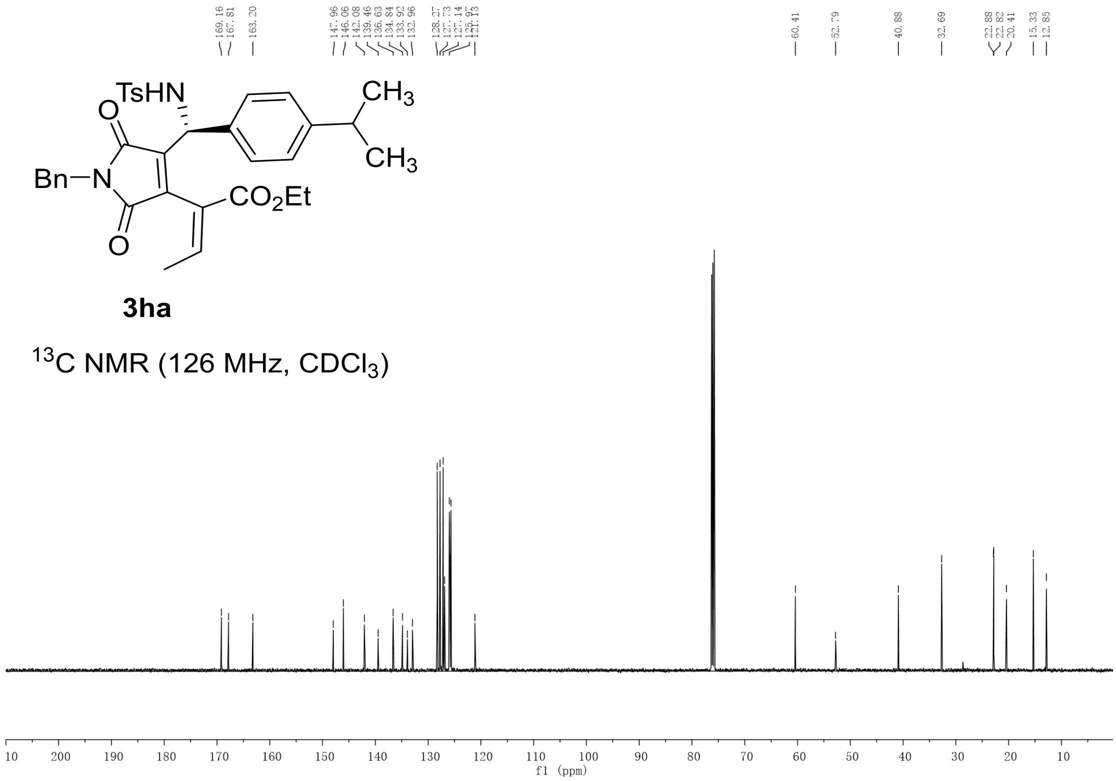
TsHN
O
Bn—N
CO2Et
O
CH3
CH3
3ha
13C NMR (126 MHz, CDCl3)
f1 (ppm)

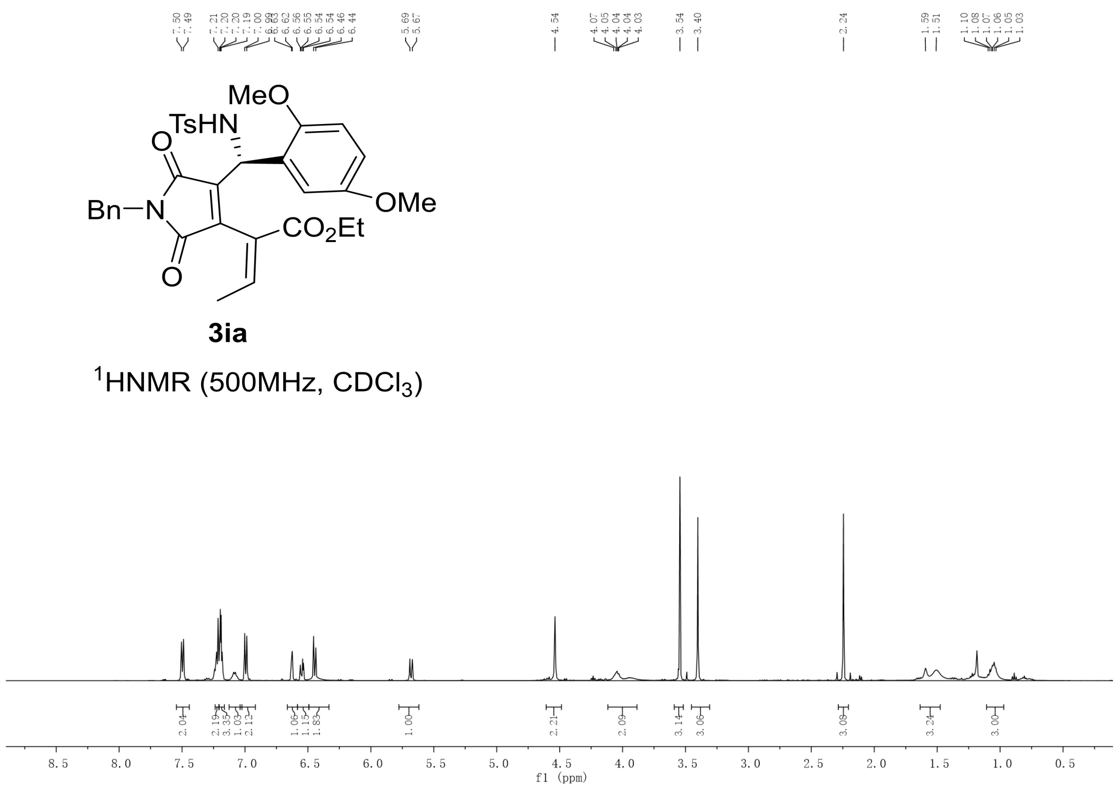
MeO
TsHN
O
Bn−N
OMe
CO2Et
O
3ia
1HNMR (500MHz, CDCl3)
f1 (ppm)

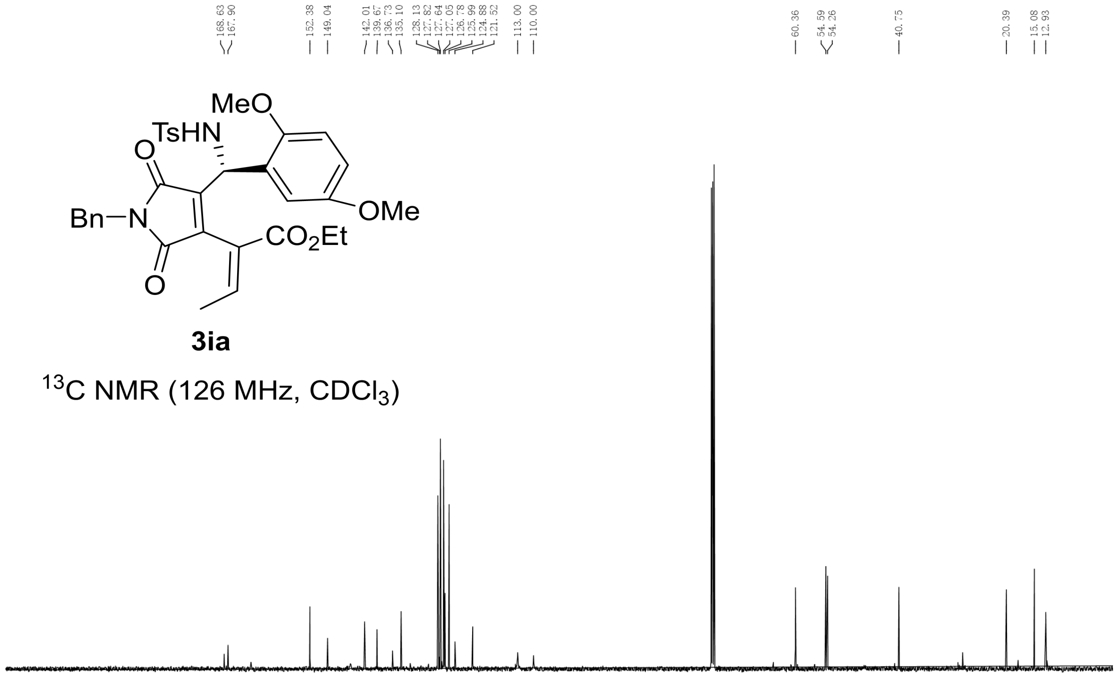
MeO
TsHN
O
Bn−N
OMe
CO2Et
O
3ia
13C NMR (126 MHz, CDCl3)
f1 (ppm)

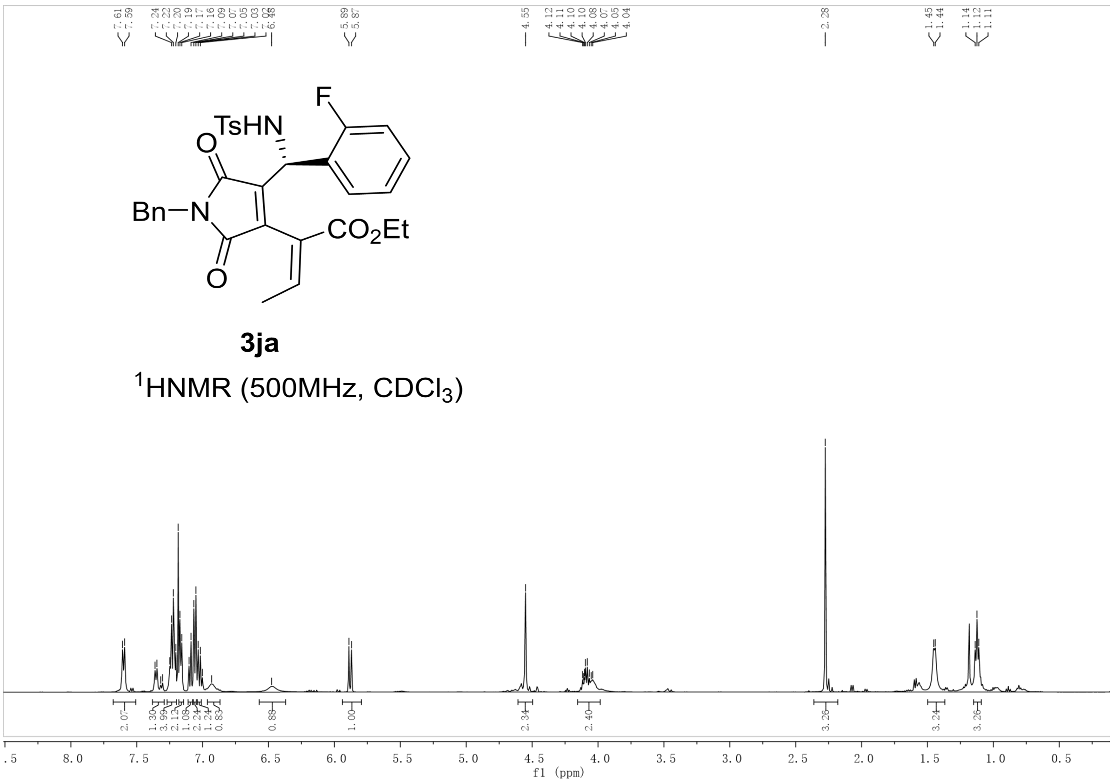

3ja

^{1}HNMR (500MHz, $CDCl_3$)

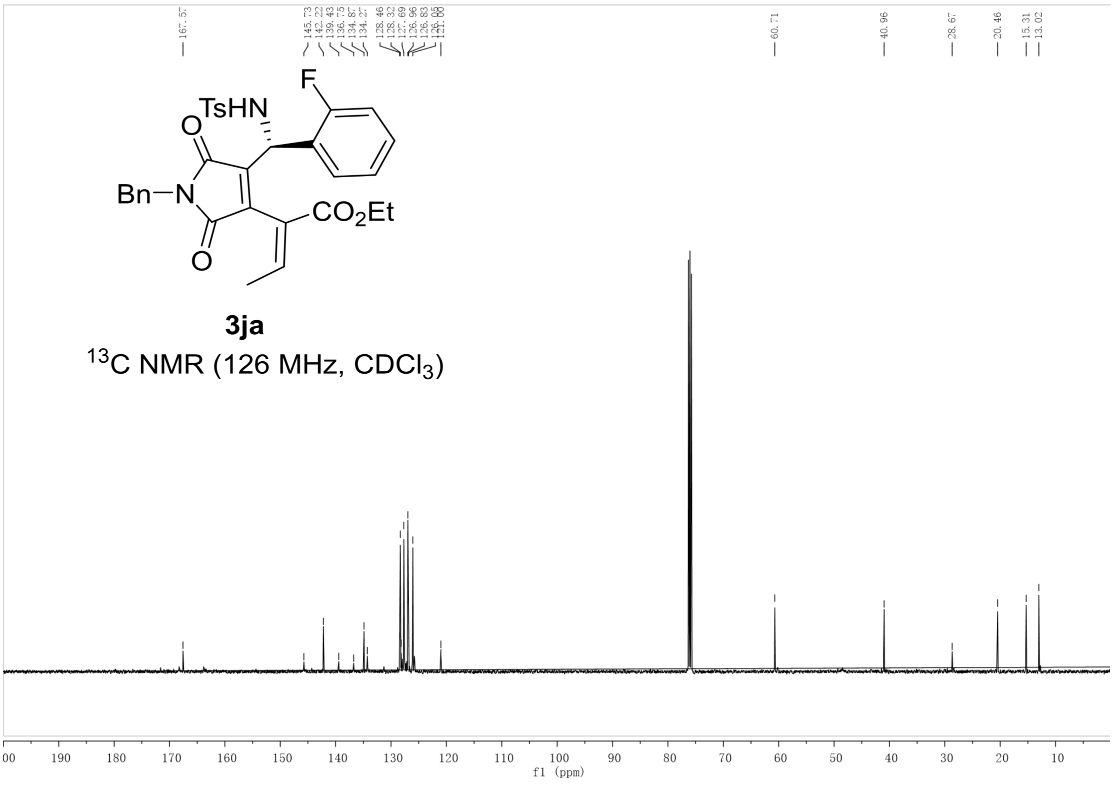

3ja

^{13}C NMR (126 MHz, $CDCl_3$)

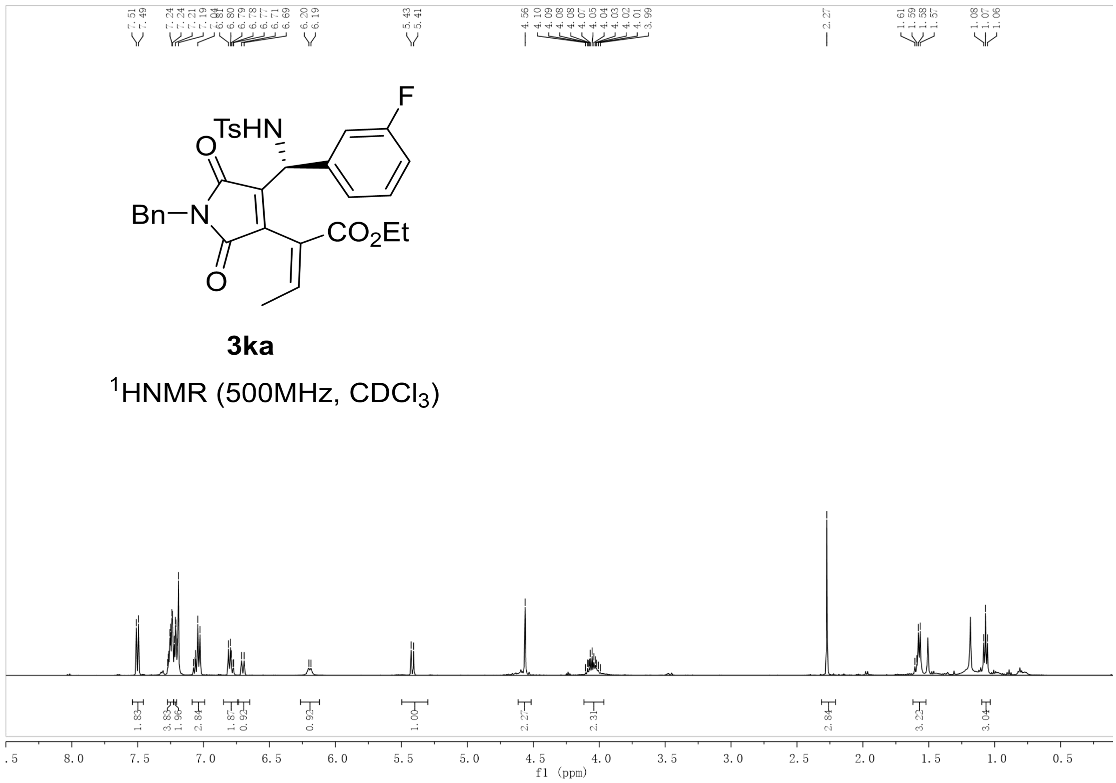
TsHN
F
O
Bn−N
CO2Et
O
3ka
1HNMR (500MHz, CDCl3)
f1 (ppm)

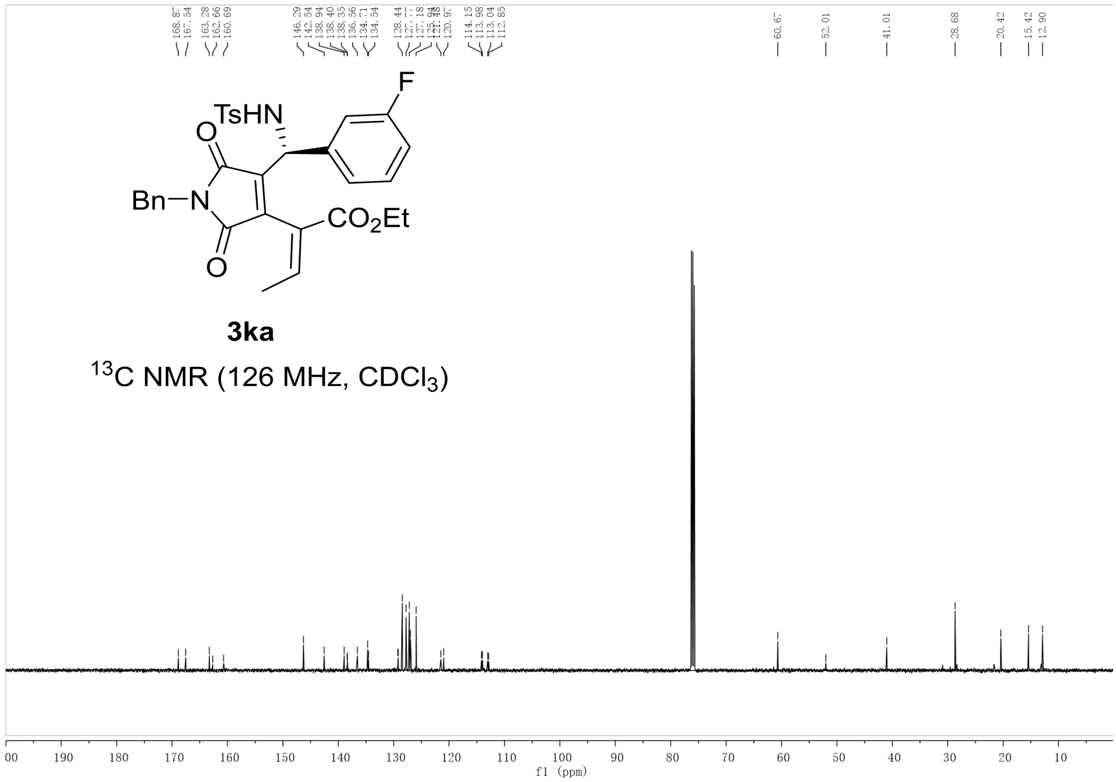
TsHN
F
O
Bn−N
CO2Et
O
3ka
13C NMR (126 MHz, CDCl3)
f1 (ppm)

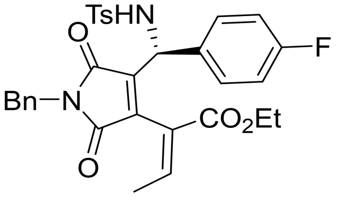

3la

^{1}HNMR (500MHz, $CDCl_3$)

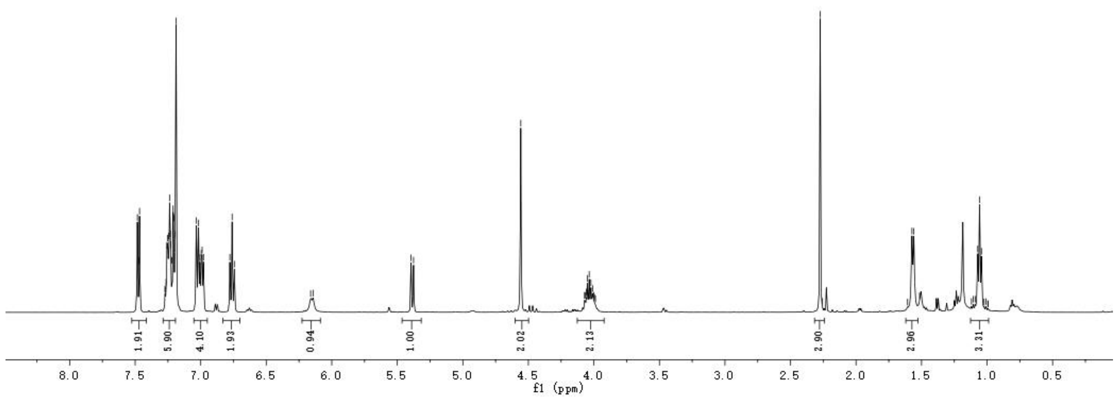

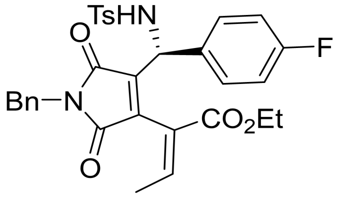

3la

^{13}C NMR (126 MHz, $CDCl_3$)

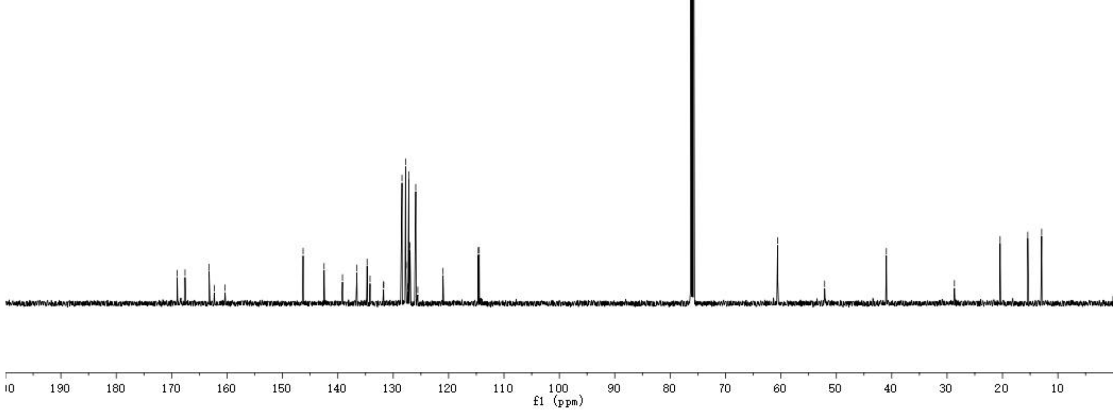

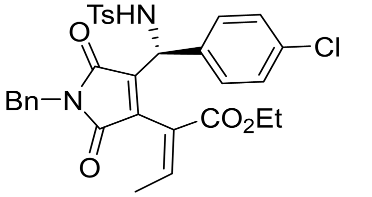

3ma

^{1}HNMR (500MHz, $CDCl_3$)

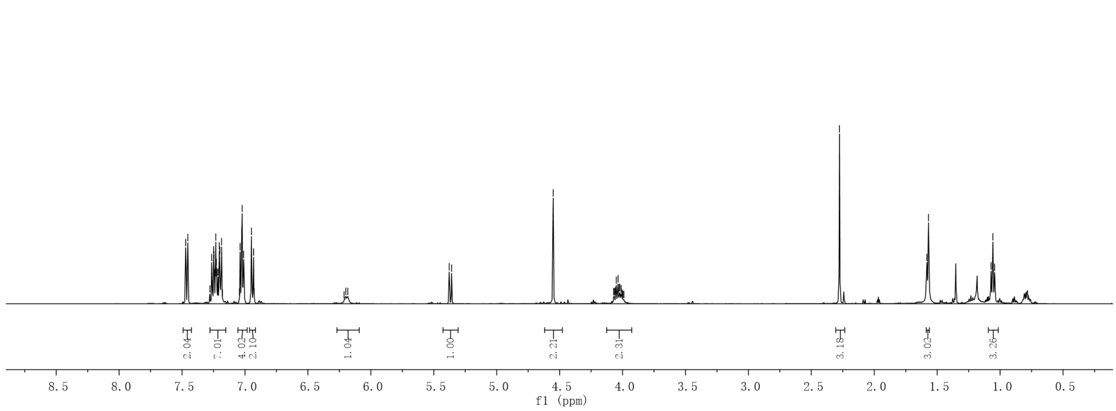

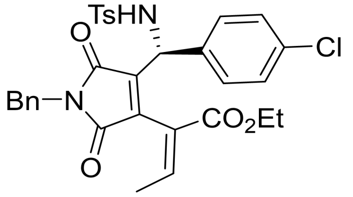

3ma

^{13}C NMR (126 MHz, $CDCl_3$)

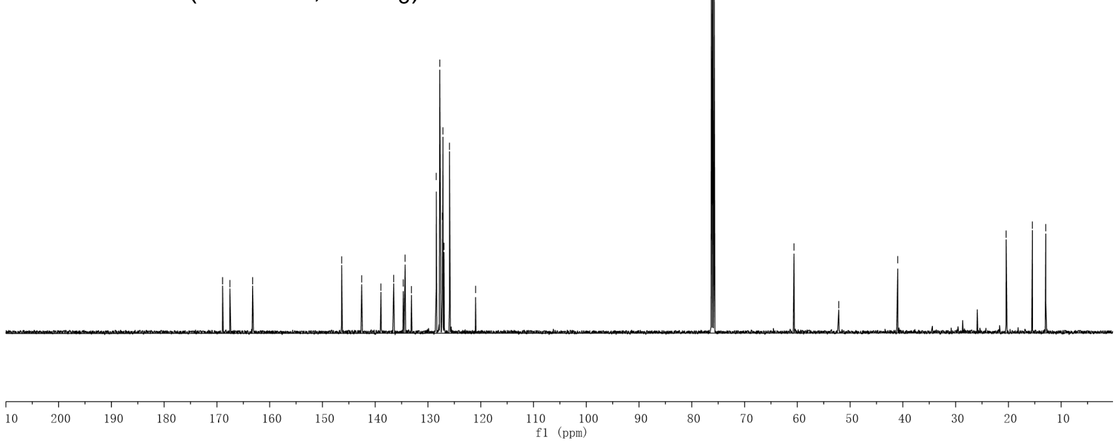

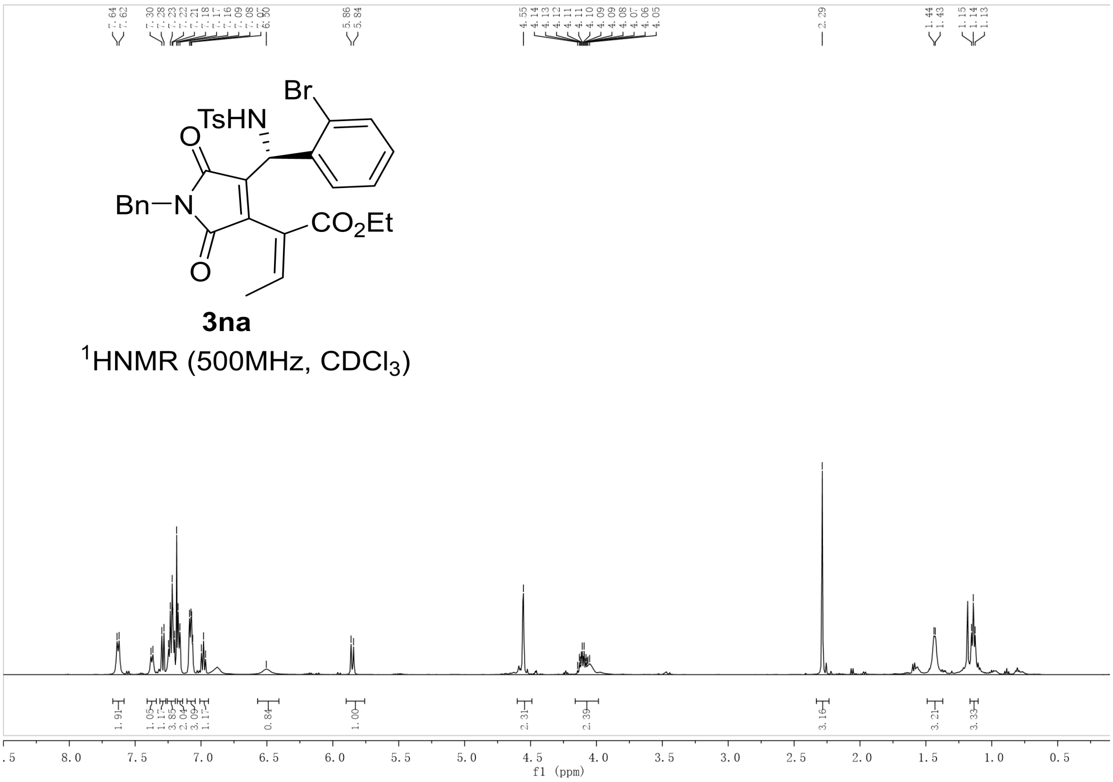

Br
TsHN
O
Bn—N
CO2Et
O
3na
1HNMR (500MHz, CDCl3)
f1 (ppm)

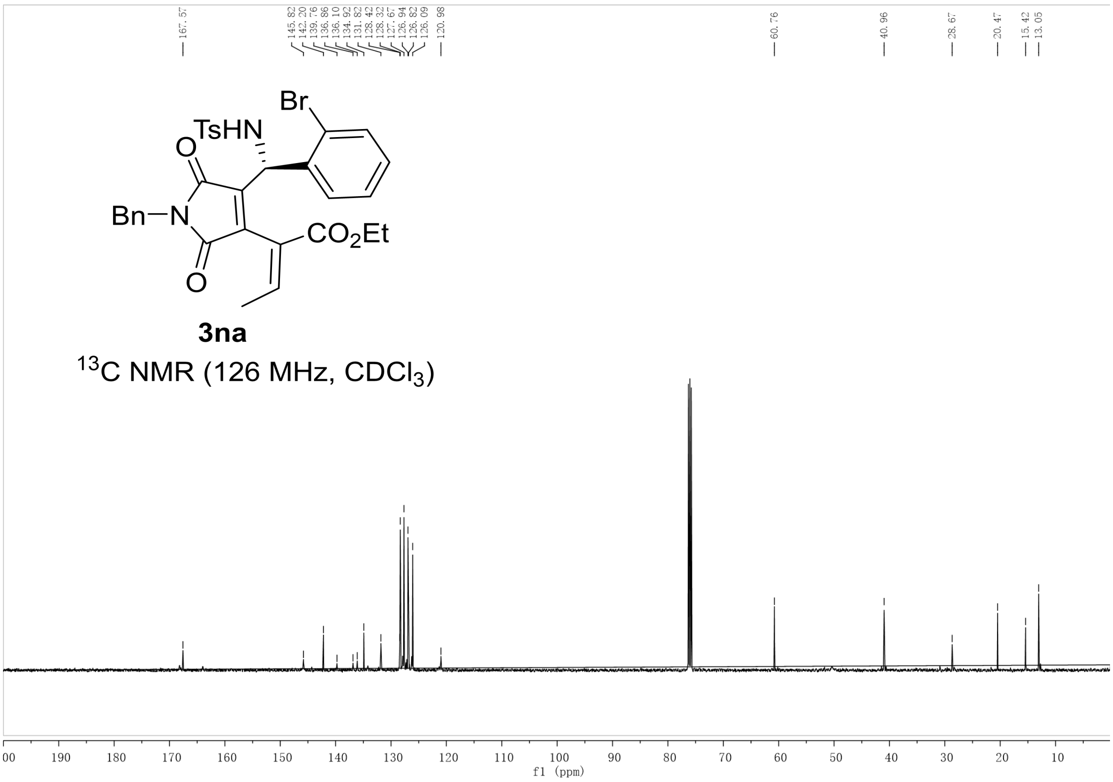

Br
TsHN
O
Bn—N
CO2Et
O
3na
13C NMR (126 MHz, CDCl3)
f1 (ppm)

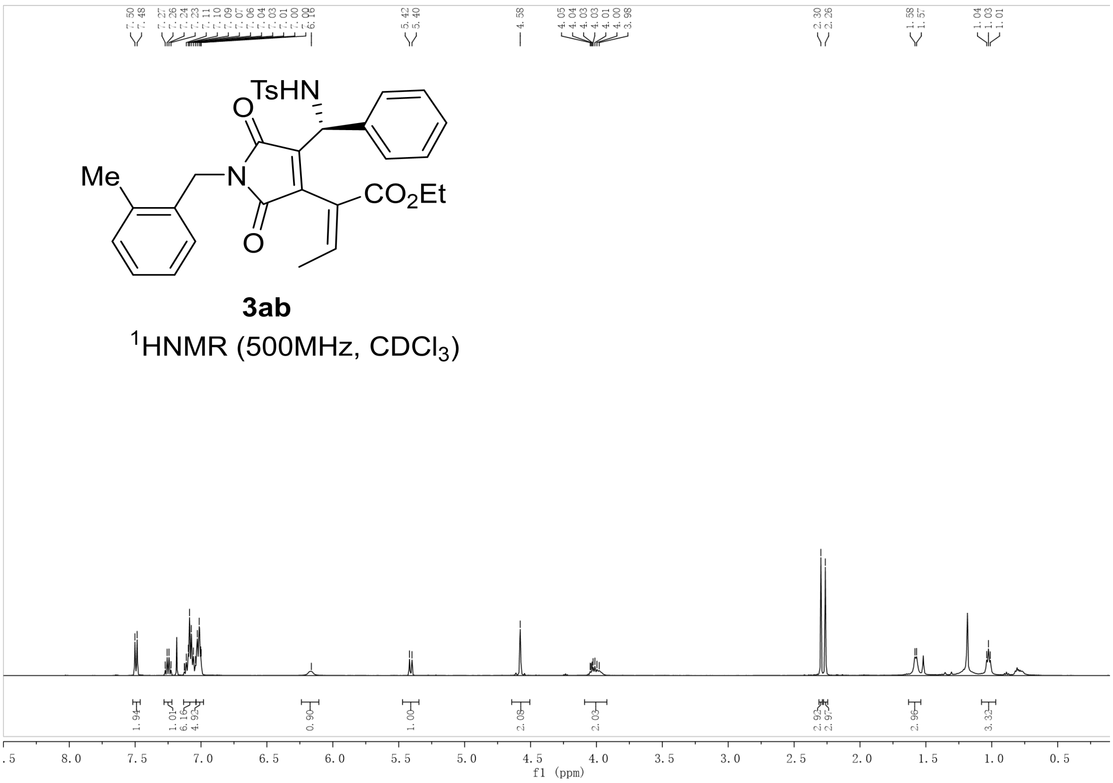
5.42
5.40
4.58
2.30
2.26
1.58
1.57
1.04
1.03
1.01
TsHN
O
Me
N
CO2Et
O
3ab
1HNMR (500MHz, CDCl3)
f1 (ppm)

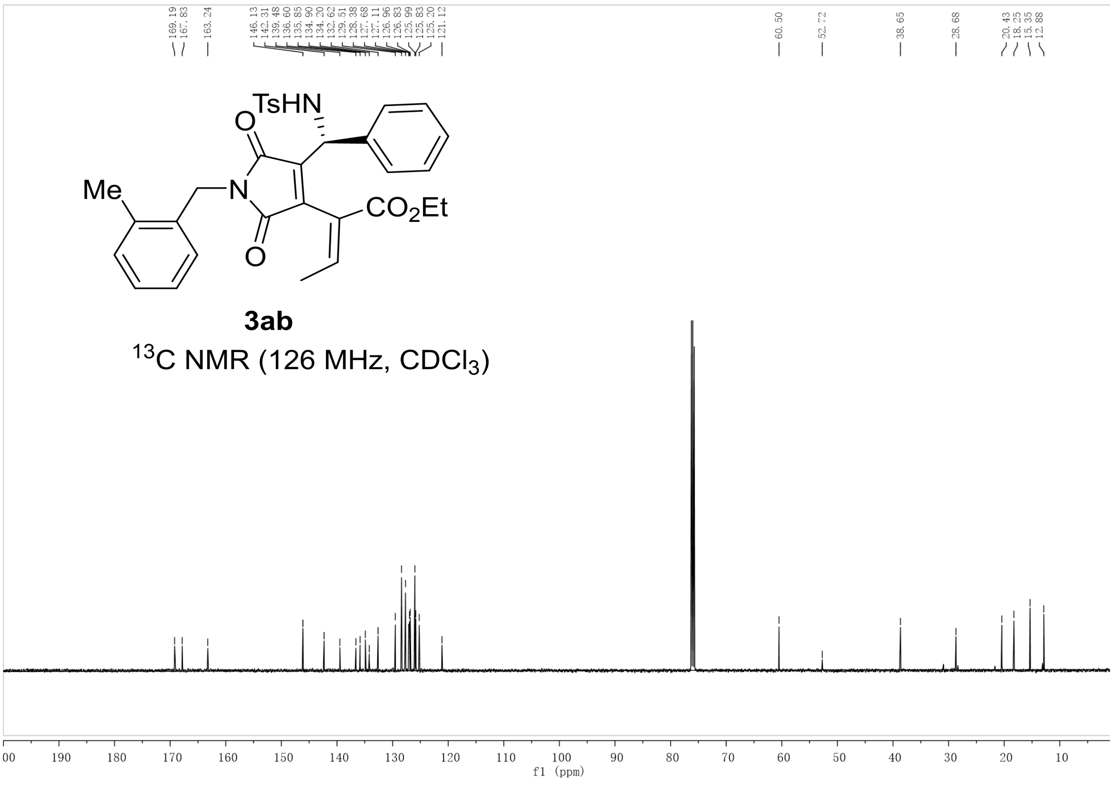
169.19
167.83
163.24
146.13
142.31
139.48
136.60
135.85
134.90
134.20
132.62
129.51
128.38
127.68
127.11
126.96
126.83
125.99
125.83
125.20
121.12
60.50
52.72
38.65
28.68
20.43
18.25
15.35
12.88
TsHN
O
Me
N
CO2Et
O
3ab
13C NMR (126 MHz, CDCl3)
f1 (ppm)

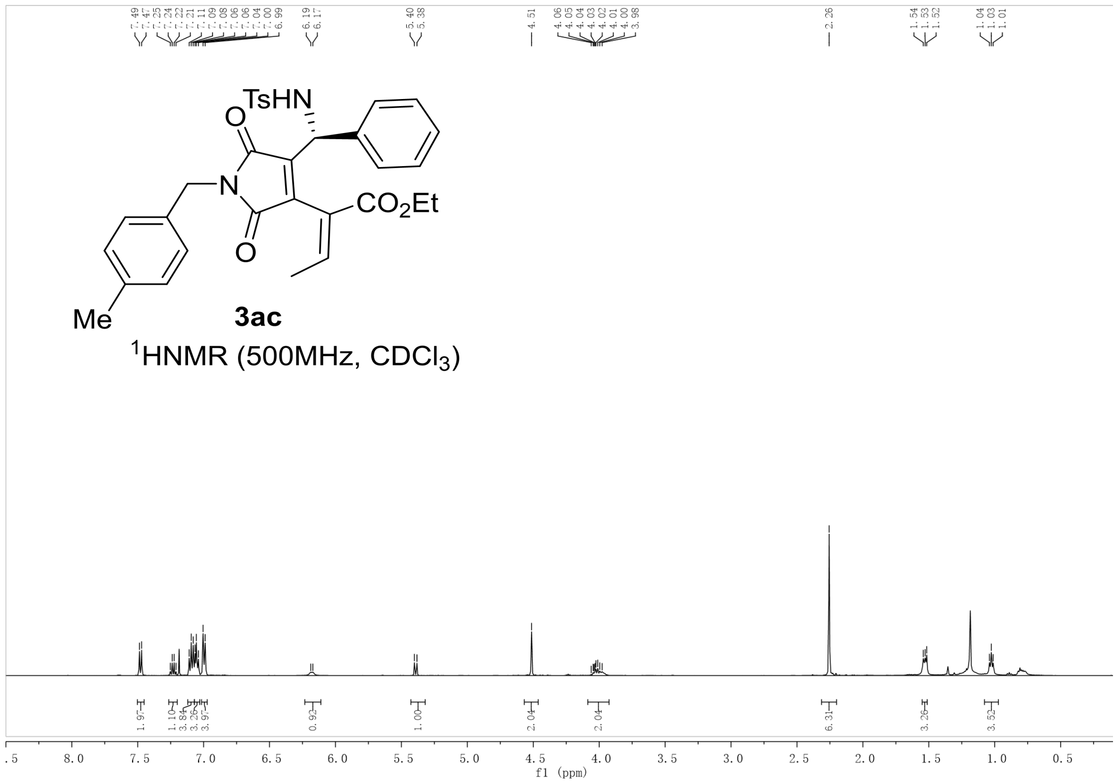
TsHN
CO2Et
Me
3ac
1HNMR (500MHz, CDCl3)
f1 (ppm)

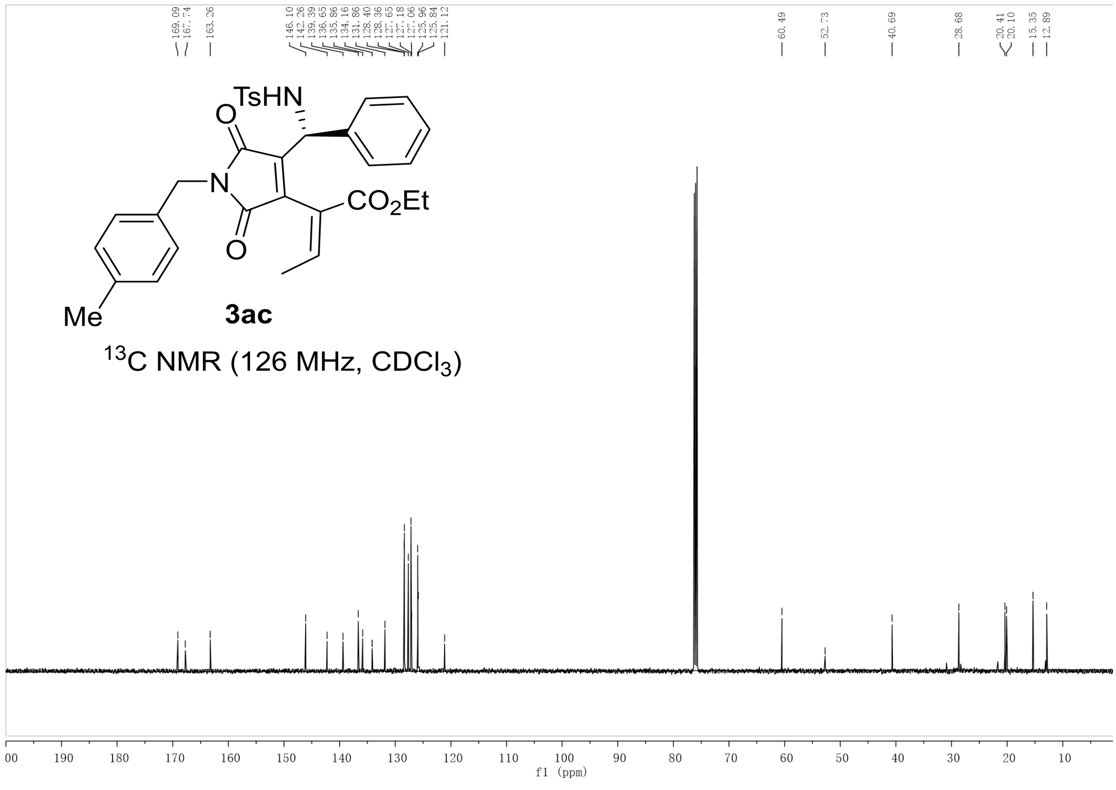
TsHN
CO2Et
Me
3ac
13C NMR (126 MHz, CDCl3)
f1 (ppm)

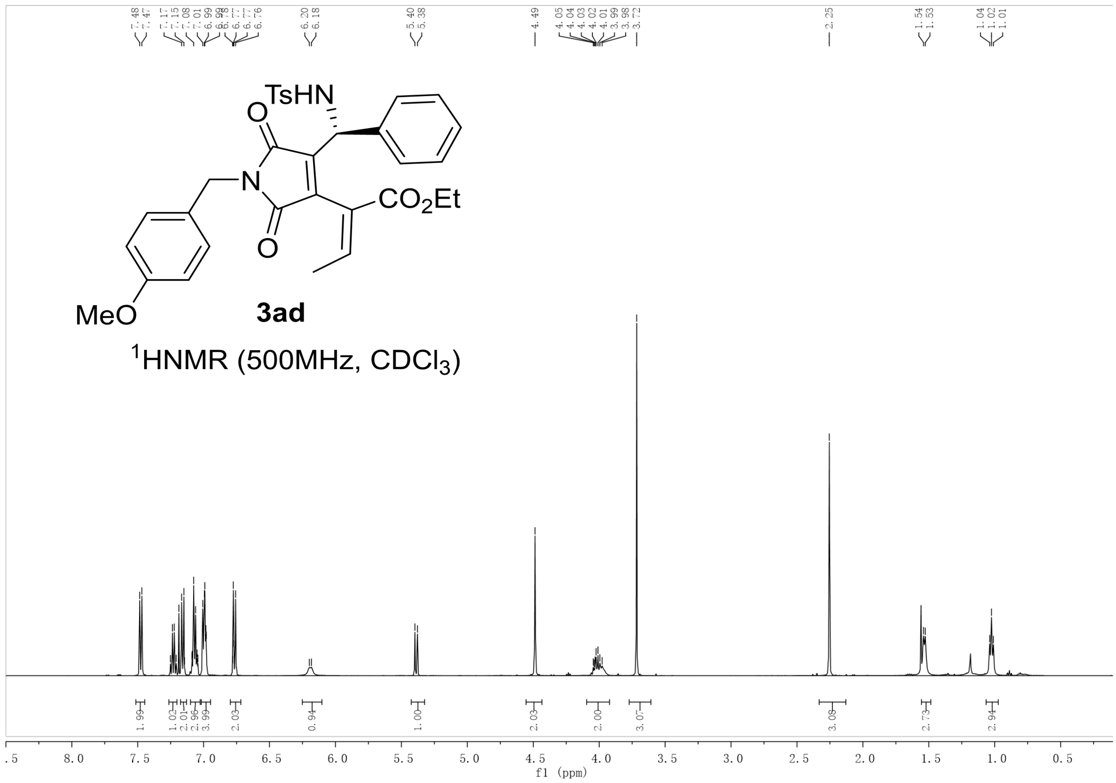
TsHN
O
N
CO2Et
O
MeO
3ad
1HNMR (500MHz, CDCl3)
f1 (ppm)

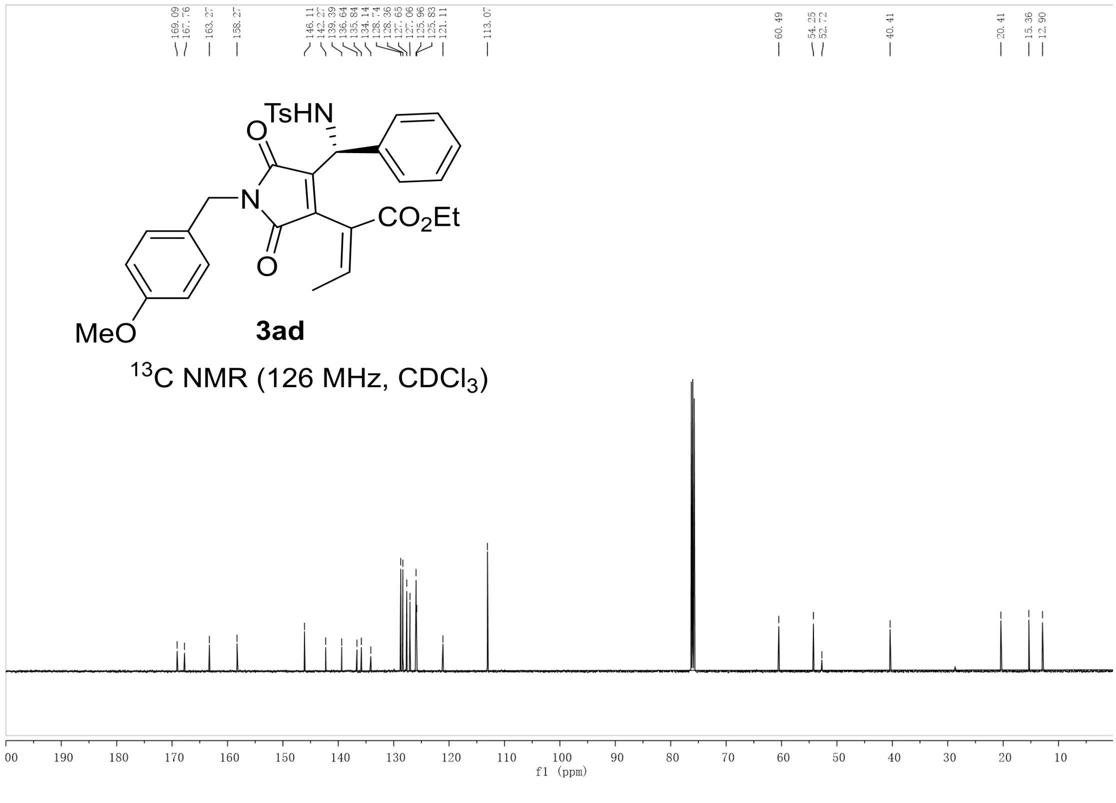
TsHN
O
N
CO2Et
O
MeO
3ad
13C NMR (126 MHz, CDCl3)
f1 (ppm)

X-Ray Crystallographic Data of 3da

Sample preparation: Single crystal suitable for X-ray diffraction was obtained by slow evaporation of a saturated solution of compound **3da** (Dichloromethane/ methanol) in a loosely capped vial.

Crystal measurement: A crystal of **3da** was picked up with paratone oil and mounted on a Bruker Smart Apexii diffractometer equipped with a Mo Kα (λ = 1.54184 Å) radiation source at 293 K.

Crystallographic data for **3da** have been deposited with the Cam-bridge Crystallographic Data Centre as deposition number CCDC 2258229.

Thermal ellipsoids are drawn at 50% probability level.

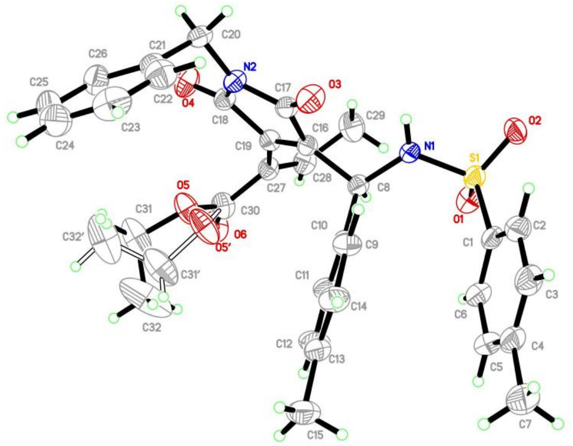

Table S1. Crystal data and structure refinement for 3da.

Identification code	**3da**
Empirical formula	$C_{32} H_{32} N_2 O_6 S$
Formula weight	572.65
Temperature/K	293
Crystal system	Monoclinic
Space group	P2(1)/n
a/Å	9.1784(3)
b/Å	12.7700(4) A
c/Å	25.7633(6) A
α/°	90
β/°	98.165(3)
γ/°	90
Volume/Å^3	2989.06(15)
Z	4

ρ_{calc} g/cm^3	1.273
μ/mm^{-1}	1.343
F(000)	1208
Crystal size/mm^3	0.120 x 0.120 x 0.110
2Θ range for data collection/°	3.466 to 67.244
Index ranges	-10<=h<=6, -14<=k<=15, -30<=l<=30
Reflections collected	10268
Independent reflections	5334 [R(int) = 0.0342]
Data/restraints/parameters	5334 / 94 / 404
Goodness-of-fit on F^2	1.052
Final R indexes [I>=2σ (I)]	R_1 = 0.0494, wR_2 = 0.1340
Final R indexes [all data]	R_1 = 0.0686, wR_2 = 0.1531
Largest diff. peak/hole / e Å^{-3}	0.295 and -0.387
Flack parameter	0.0031(3)

Table S2 Fractional Atomic Coordinates (×10^4) and Equivalent Isotropic Displacement Parameters (Å^2×10^3) for 3da. U_{eq} is defined as 1/3 of of the trace of the orthogonalised U_{IJ} tensor.

Atom	x	y	z	U(eq)
S(1)	7648(1)	9580(1)	931(1)	50(1)
N(1)	7253(2)	8545(2)	1255(1)	43(1)
N(2)	5862(2)	6036(2)	2193(1)	47(1)
O(1)	6342(2)	10205(1)	852(1)	66(1)
O(2)	8979(2)	9990(2)	1214(1)	69(1)
O(3)	7802(2)	6390(2)	1743(1)	62(1)
O(4)	3637(2)	6193(2)	2496(1)	72(1)
O(5)	1931(8)	6202(6)	1394(3)	74(2)
C(31)	766(9)	5458(7)	1249(3)	110(3)
C(32)	468(10)	5417(9)	667(3)	179(5)
O(5')	2050(20)	6394(16)	1187(7)	93(4)
C(31')	910(20)	5727(13)	919(9)	115(5)
C(32')	610(30)	4839(19)	1277(9)	178(7)
O(6)	325(2)	7532(2)	1338(1)	79(1)
C(1)	8036(3)	9181(2)	310(1)	49(1)
C(2)	9428(3)	8832(2)	258(1)	61(1)
C(3)	9695(3)	8447(2)	-221(1)	69(1)
C(4)	8591(4)	8399(2)	-644(1)	65(1)
C(5)	7228(3)	8770(3)	-584(1)	67(1)
C(6)	6931(3)	9166(2)	-114(1)	58(1)
C(7)	8892(4)	7943(3)	-1160(1)	95(1)
C(8)	5813(2)	8032(2)	1118(1)	42(1)

C(9)	5634(3)	7387(2)	616(1)	44(1)
C(10)	6745(3)	6762(2)	485(1)	54(1)
C(11)	6570(3)	6209(2)	23(1)	64(1)
C(12)	5291(3)	6245(2)	-324(1)	62(1)
C(13)	4171(3)	6864(3)	-191(1)	74(1)
C(14)	4346(3)	7433(3)	272(1)	64(1)
C(15)	5118(4)	5640(3)	-834(1)	86(1)
C(16)	5508(2)	7364(2)	1574(1)	40(1)
C(17)	6566(3)	6564(2)	1828(1)	44(1)
C(18)	4486(3)	6455(2)	2202(1)	49(1)
C(19)	4270(2)	7293(2)	1788(1)	43(1)
C(20)	6555(3)	5216(2)	2540(1)	56(1)
C(21)	6381(3)	4147(2)	2302(1)	50(1)
C(22)	7463(3)	3708(3)	2047(1)	69(1)
C(23)	7259(4)	2717(3)	1818(1)	86(1)
C(24)	5997(5)	2175(3)	1847(1)	87(1)
C(25)	4949(4)	2585(3)	2106(1)	86(1)
C(26)	5139(3)	3555(2)	2330(1)	68(1)
C(27)	2862(3)	7851(2)	1656(1)	48(1)
C(28)	2681(3)	8869(2)	1724(1)	59(1)
C(29)	3831(4)	9624(2)	1947(1)	81(1)
C(30)	1566(3)	7221(2)	1433(1)	62(1)

Table S3. Anisotropic displacement parameters (A^2 x 10^3) for 3da. The anisotropic displacement factor exponent takes the form: -2 pi^2 [h^2 a*^2 U11+ ... + 2 h k a* b* U12]

Atom	U11	U22	U33	U23	U13	U12
S(1)	65(1)	47(1)	43(1)	-4(1)	19(1)	-9(1)
N(1)	43(1)	52(1)	35(1)	4(1)	7(1)	-7(1)
N(2)	51(1)	49(1)	42(1)	7(1)	8(1)	1(1)
O(1)	84(1)	51(1)	67(1)	4(1)	28(1)	12(1)
O(2)	81(1)	76(1)	53(1)	-16(1)	18(1)	-37(1)
O(3)	40(1)	80(1)	68(1)	17(1)	13(1)	14(1)
O(4)	74(1)	80(1)	69(1)	16(1)	37(1)	-2(1)
O(5)	45(2)	65(3)	111(4)	-22(3)	13(2)	-6(2)
C(31)	71(4)	82(5)	178(7)	-39(5)	17(5)	-15(4)
C(32)	96(5)	218(9)	210(9)	-134(8)	-21(6)	21(6)
O(5')	44(5)	89(8)	145(10)	-43(8)	13(7)	-13(5)
C(31')	66(7)	106(9)	169(11)	-52(9)	4(9)	-6(7)
C(32')	152(13)	122(13)	264(16)	-48(14)	41(13)	-51(12)
O(6)	37(1)	85(2)	115(2)	7(1)	13(1)	6(1)
C(1)	60(2)	49(1)	39(1)	2(1)	18(1)	-7(1)
C(2)	57(2)	77(2)	50(1)	0(1)	14(1)	-6(2)

C(3)	68(2)	78(2)	66(2)	-4(2)	30(1)	-4(2)
C(4)	83(2)	68(2)	48(1)	-4(1)	25(1)	-14(2)
C(5)	76(2)	81(2)	43(1)	6(1)	6(1)	-9(2)
C(6)	64(2)	66(2)	46(1)	8(1)	12(1)	2(1)
C(7)	123(3)	106(3)	65(2)	-24(2)	41(2)	-24(2)
C(8)	36(1)	47(1)	42(1)	4(1)	7(1)	1(1)
C(9)	46(1)	49(1)	39(1)	6(1)	6(1)	-4(1)
C(10)	52(2)	62(2)	46(1)	-3(1)	1(1)	9(1)
C(11)	75(2)	64(2)	54(1)	-4(1)	9(1)	11(2)
C(12)	82(2)	60(2)	42(1)	1(1)	4(1)	-7(2)
C(13)	63(2)	99(2)	54(2)	-4(2)	-11(1)	-3(2)
C(14)	50(2)	85(2)	53(1)	0(1)	0(1)	5(2)
C(15)	126(3)	81(2)	49(2)	-9(2)	-1(2)	-7(2)
C(16)	38(1)	44(1)	38(1)	-1(1)	7(1)	-1(1)
C(17)	44(1)	47(1)	41(1)	0(1)	4(1)	0(1)
C(18)	54(2)	50(1)	43(1)	-1(1)	13(1)	-4(1)
C(19)	41(1)	44(1)	43(1)	-4(1)	10(1)	1(1)
C(20)	69(2)	54(2)	41(1)	8(1)	-2(1)	0(1)
C(21)	60(2)	51(1)	37(1)	9(1)	-1(1)	4(1)
C(22)	59(2)	78(2)	70(2)	4(2)	7(1)	8(2)
C(23)	97(3)	82(2)	79(2)	-1(2)	16(2)	34(2)
C(24)	114(3)	55(2)	85(2)	-2(2)	-6(2)	8(2)
C(25)	96(3)	61(2)	98(2)	4(2)	5(2)	-17(2)
C(26)	73(2)	62(2)	72(2)	7(2)	18(2)	-7(2)
C(27)	42(1)	55(2)	50(1)	2(1)	16(1)	0(1)
C(28)	50(2)	62(2)	69(2)	5(1)	20(1)	7(1)
C(29)	83(2)	60(2)	103(2)	-11(2)	20(2)	1(2)
C(30)	45(2)	69(2)	75(2)	-1(2)	17(1)	-1(1)

Table S4 Bond Lengths for 3da.

Atom-Atom	Length/Å	Atom-Atom	Length/Å
S(1)-O(1)	1.4301(19)	S(1)-O(2)	1.4305(18)
S(1)-N(1)	1.6306(19)	S(1)-C(1)	1.764(2)
N(1)-C(8)	1.473(3)	N(2)-C(18)	1.374(3)
N(2)-C(17)	1.387(3)	N(2)-C(20)	1.464(3)
O(3)-C(17)	1.207(3)	O(4)-C(18)	1.209(3)
O(5)-C(30)	1.351(8)	O(5)-C(31)	1.439(6)
C(31)-C(32)	1.486(8)	O(5')-C(30)	1.341(19)
O(5')-C(31')	1.449(9)	C(31')-C(32')	1.512(10)
O(6)-C(30)	1.198(3)	C(1)-C(2)	1.377(4)
C(1)-C(6)	1.380(3)	C(2)-C(3)	1.383(4)
C(3)-C(4)	1.380(4)	C(4)-C(5)	1.367(4)

C(4)-C(7)	1.512(4)	C(5)-C(6)	1.375(4)
C(8)-C(16)	1.509(3)	C(8)-C(9)	1.522(3)
C(9)-C(14)	1.374(3)	C(9)-C(10)	1.374(3)
C(10)-C(11)	1.373(3)	C(11)-C(12)	1.371(4)
C(12)-C(13)	1.378(4)	C(12)-C(15)	1.513(4)
C(13)-C(14)	1.387(4)	C(16)-C(19)	1.335(3)
C(16)-C(17)	1.495(3)	C(18)-C(19)	1.505(3)
C(19)-C(27)	1.472(3)	C(20)-C(21)	1.497(4)
C(21)-C(26)	1.378(4)	C(21)-C(22)	1.385(4)
C(22)-C(23)	1.397(5)	C(23)-C(24)	1.360(5)
C(24)-C(25)	1.350(5)	C(25)-C(26)	1.369(5)
C(27)-C(28)	1.326(4)	C(27)-C(30)	1.482(4)
C(28)-C(29)	1.484(4)		

Table S5 Bond Angles for 3da.

Atom-Atom-Atom	Angle/°	Atom-Atom-Atom	Angle/°
O(1)-S(1)-O(2)	120.44(13)	O(1)-S(1)-N(1)	106.41(11)
O(2)-S(1)-N(1)	105.73(11)	O(1)-S(1)-C(1)	107.74(12)
O(2)-S(1)-C(1)	107.50(12)	N(1)-S(1)-C(1)	108.57(11)
C(8)-N(1)-S(1)	119.68(14)	C(18)-N(2)-C(17)	109.92(19)
C(18)-N(2)-C(20)	126.0(2)	C(17)-N(2)-C(20)	123.9(2)
C(30)-O(5)-C(31)	118.2(6)	O(5)-C(31)-C(32)	108.0(10)
C(30)-O(5')-C(31')	114.8(15)	O(5')-C(31')-C(32')	109(2)
C(2)-C(1)-C(6)	120.4(2)	C(2)-C(1)-S(1)	119.41(19)
C(6)-C(1)-S(1)	120.1(2)	C(1)-C(2)-C(3)	119.1(3)
C(4)-C(3)-C(2)	121.2(3)	C(5)-C(4)-C(3)	118.4(3)
C(5)-C(4)-C(7)	121.2(3)	C(3)-C(4)-C(7)	120.4(3)
C(4)-C(5)-C(6)	121.8(3)	C(5)-C(6)-C(1)	119.1(3)
N(1)-C(8)-C(16)	108.91(16)	N(1)-C(8)-C(9)	115.42(18)
C(16)-C(8)-C(9)	110.23(19)	C(14)-C(9)-C(10)	117.8(2)
C(14)-C(9)-C(8)	120.2(2)	C(10)-C(9)-C(8)	121.9(2)
C(11)-C(10)-C(9)	120.8(2)	C(12)-C(11)-C(10)	122.1(3)
C(11)-C(12)-C(13)	117.2(3)	C(11)-C(12)-C(15)	121.5(3)
C(13)-C(12)-C(15)	121.3(3)	C(12)-C(13)-C(14)	121.1(3)
C(9)-C(14)-C(13)	121.0(3)	C(19)-C(16)-C(17)	108.08(19)
C(19)-C(16)-C(8)	128.5(2)	C(17)-C(16)-C(8)	123.14(19)
O(3)-C(17)-N(2)	125.1(2)	O(3)-C(17)-C(16)	127.9(2)
N(2)-C(17)-C(16)	107.02(19)	O(4)-C(18)-N(2)	125.4(2)
O(4)-C(18)-C(19)	127.5(2)	N(2)-C(18)-C(19)	107.03(19)
C(16)-C(19)-C(27)	130.0(2)	C(16)-C(19)-C(18)	107.9(2)
C(27)-C(19)-C(18)	122.1(2)	N(2)-C(20)-C(21)	112.93(19)
C(26)-C(21)-C(22)	117.3(3)	C(26)-C(21)-C(20)	121.3(3)

C(22)-C(21)-C(20)	121.5(3)	C(21)-C(22)-C(23)	120.3(3)
C(24)-C(23)-C(22)	120.1(3)	C(25)-C(24)-C(23)	120.1(3)
C(24)-C(25)-C(26)	120.2(3)	C(25)-C(26)-C(21)	122.0(3)
C(28)-C(27)-C(19)	124.5(2)	C(28)-C(27)-C(30)	118.4(2)
C(19)-C(27)-C(30)	117.2(2)	C(27)-C(28)-C(29)	126.4(3)
O(6)-C(30)-O(5')	122.4(8)	O(6)-C(30)-O(5)	122.9(4)
O(6)-C(30)-C(27)	125.9(3)	O(5')-C(30)-C(27)	107.8(7)
O(5)-C(30)-C(27)	111.0(4)		

Table S6 Hydrogen Atom Coordinates (Å×10^4) and Isotropic Displacement Parameters (Å^2×10^3) for 3da.

Atom	x	y	z	U(eq)
H(1)	7883	8300	1504	52
H(31A)	-114	5673	1389	132
H(31B)	1057	4773	1388	132
H(32A)	278	6111	531	268
H(32B)	-375	4982	561	268
H(32C)	1307	5131	533	268
H(31C)	1218	5448	602	138
H(31D)	19	6133	821	138
H(32D)	204	5116	1573	267
H(32E)	1508	4477	1397	267
H(32F)	-85	4362	1089	267
H(2)	10178	8854	541	73
H(3)	10635	8216	-259	82
H(5)	6482	8755	-868	80
H(6)	5999	9420	-82	70
H(7A)	8137	7446	-1284	142
H(7B)	9831	7600	-1111	142
H(7C)	8898	8495	-1413	142
H(8)	5066	8585	1072	50
H(10)	7626	6712	712	65
H(11)	7343	5797	-57	77
H(13)	3284	6900	-415	89
H(14)	3580	7853	352	76
H(15A)	4090	5553	-961	130
H(15B)	5571	4965	-775	130
H(15C)	5581	6017	-1088	130
H(20A)	6126	5221	2863	67
H(20B)	7596	5371	2628	67
H(22)	8329	4074	2027	83
H(23)	7987	2427	1646	103

H(24)	5855	1522	1689	104
H(25)	4097	2206	2131	103
H(26)	4407	3824	2508	82
H(28)	1742	9136	1622	71
H(29A)	4717	9252	2077	122
H(29B)	3494	10003	2229	122
H(29C)	4025	10107	1679	122

Ellipsoid Plot of **3da**

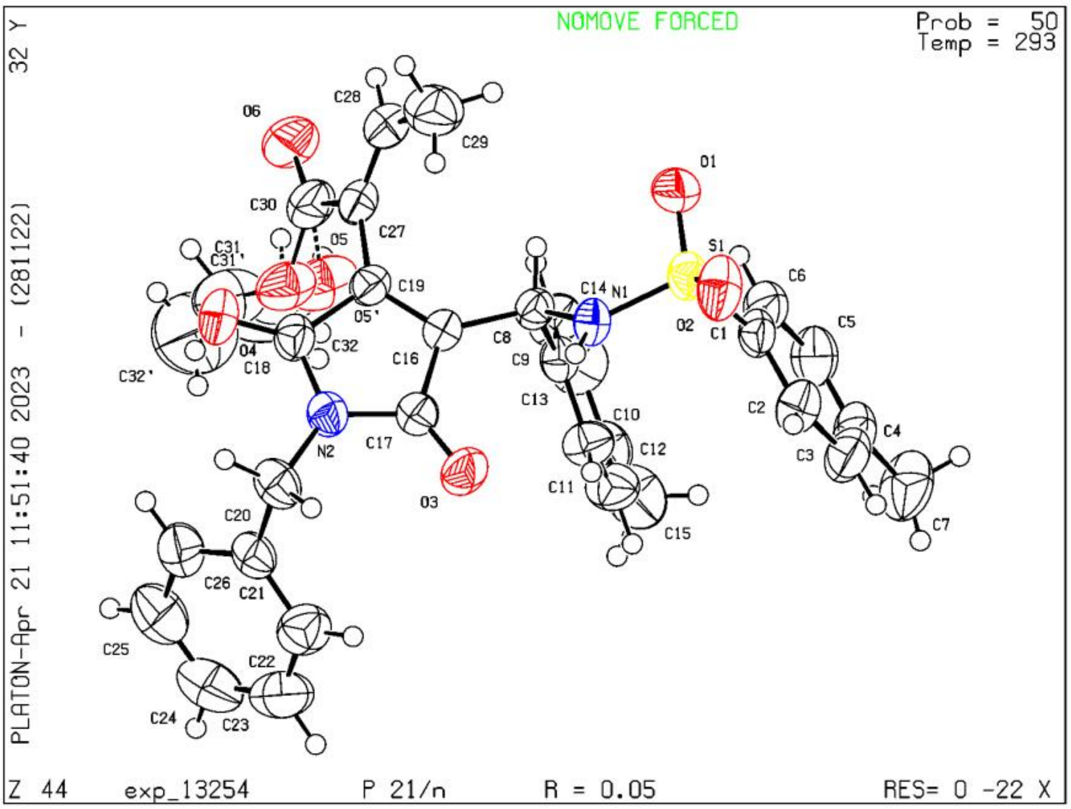

Supplement: Supplementary file 1 [file ijms-25-06916-s001.zip › ijms-3077080-supplementary.pdf]
